# Herpes Simplex Virus 1 Manipulates Host Cell Antiviral and Proviral DNA Damage Responses

**DOI:** 10.1128/mBio.03552-20

**Published:** 2021-02-09

**Authors:** Max E. Mertens, David M. Knipe

**Affiliations:** a Department of Microbiology, Blavatnik Institute, Harvard Medical School, Boston, Massachusetts, USA; b Program in Virology, Harvard Medical School, Boston, Massachusetts, USA; Duke University Medical Center

**Keywords:** DNA damage response, herpes simplex virus, viral replication

## Abstract

Cells activate their DNA damage response (DDR) in response to DNA virus infection, including adenoviruses, papillomaviruses, polyomaviruses, and herpesviruses. In this study, we found that the DDR kinase pathways activated in normal human fibroblasts by herpes simplex virus 1 (HSV-1) input genomic DNA, HSV-1 replicating DNA, and progeny DNA and in uninfected cells treated with etoposide are different. We also found using clustered regularly interspaced palindromic repeat (CRISPR)-Cas9 technology that different host gene products are required for the DDR in uninfected versus infected cells. Individual DDR components can be proviral or antiviral in that ataxia-telangiectasia mutated (ATM) and p53 promote and Mre11 restricts replication of ICP0-null HSV-1, but ICP0 expression eliminates these DDR effects. Thus, in total, these results argue that HSV-1 manipulates the host cell DDR to utilize specific components for its optimal replication while inactivating the antiviral aspects of the DDR.

## Abstract (precis)

We investigated the relationship between the DNA damage response, a collection of vital cellular pathways that repair potentially lethal damage to the genome, and the DNA virus herpes simplex virus 1. We found that infection by the virus triggers the DNA damage response and key proteins that mediate this response have opposing effects on the replication and production of progeny viruses.

## INTRODUCTION

Cells have evolved complex pathways of machinery with the purpose of repairing breaks in the DNA to preserve the integrity of the genome. The Mre11-Rad50-Nbs1 (MRN) complex is responsible for first sensing and then binding to double-stranded DNA ends (1). This complex is crucial for the recruitment of a key DNA damage response (DDR) kinase, ataxia-telangiectasia mutated (ATM), via an interaction with Nbs1 (2). In response to DNA damage, ATM phosphorylates the H2AX histone variant (3), which is commonly used as a marker of DNA damage. Phosphorylated H2AX, termed γH2AX, is then bound by the DDR adapter protein Mdc1 to generate a signaling platform to recruit additional repair factors, including the RNF8 and RNF168 E3 ubiquitin ligases, which are required for recruitment of 53BP1 to damage loci (4–7). This pathway promotes homologous recombination (HR) of complementary DNA sequences for repair of the double-stranded DNA breaks.

Double-stranded breaks are also repaired using the nonhomologous-end-joining (NHEJ) pathway. Free double-stranded DNA ends are first bound by the heterodimeric Ku70/Ku80 complex, which then recruits the catalytic subunit of DNA-dependent protein kinase (DNA-PKcs) (8, 9). Through the interactions between a complex of proteins, the free DNA ends are tethered and ligated by the XRCC4-DNA ligase IV (LIG4) complex (10–12).

The p53 tumor suppressor protein is activated to regulate cellular gene expression to allow the repair of the damage or to induce death of the cell by apoptosis (13). Two key residues that are phosphorylated in response to DNA damage are serines 15 and 20, predominantly phosphorylated by ATM/ATR and Chk2, respectively (14–16). Phosphorylation of serine 15 of p53 has been suggested to regulate the transcriptional transactivation activity of p53 (17), and serine 20 the turnover of the protein (18).

The genomes of DNA viruses do not go unnoticed by the DDR. High-risk human papillomaviruses (HPVs) use DDR pathways to efficiently replicate their genomes. HPV upregulates expression of the MRN complex (19) and promotes the activation of ATM to efficiently amplify the viral genome (20). In contrast, human adenovirus promotes both the mislocalization and degradation of the MRN complex (21), which along with ATM restricts replication of the virus (22). The human herpesvirus Epstein-Barr virus (EBV) downregulates the ATM pathway (23), while another human herpesvirus, Kaposi’s sarcoma-associated herpesvirus (KSHV), relies on the functions of Mre11 and ATM for optimal replication (24). A third human herpesvirus, human cytomegalovirus (HCMV), also relies on ATM for efficient replication (25). The differing roles of the DDR in the life cycles of these DNA viruses underscore how viruses manipulate the host cell to optimize their replication processes.

Herpes simplex virus 1 (HSV-1) is another large, double-stranded DNA virus with a genome of approximately 152 kbp. Shortly following the docking of the viral capsid at the nuclear pore, the linear viral genome is released into the nucleoplasm, where it rapidly circularizes and begins transcription of a characteristic cascade of three kinetic classes of genes (26). The first is the immediate early (IE) class, consisting of five genes: *ICP0*, *ICP4*, *ICP22*, *ICP27*, and *ICP47*. The ICP0 protein plays a central role in targeting host restriction factors for proteasome-mediated degradation using its E3 ubiquitin ligase activity (27–31). ICP4 activates transcription of the early (E) gene class, encoding primarily the core machinery necessary to replicate the viral genome along with other miscellaneous proteins involved in optimizing the replication process (26). Expressed following DNA replication, the viral late (L) gene class encodes gene products that package the replicated genomes and assemble new particles (26).

Although at least part of the input HSV-1 DNA is circularized, the host cell still initiates a response to double-stranded DNA breaks. The homologous recombination (HR) pathway is activated soon after infection, and HR proteins localize adjacent to incoming viral genomes and the eventual viral DNA replication centers (32–34). While both ATM and the related ATR DDR kinase appear to be activated by infection, it is unclear whether they affect replication. ATM activity has been shown to promote HSV-1 gene expression and replication in some infection conditions (32, 35) but is dispensable in others (36–38). ATR has also been reported to promote replication (38), but another study reported that infection causes the aberrant localization of this arm of the HR pathway (39). Indeed, various DDR proteins associate with the viral DNA and localize to viral replication compartments (32, 34, 37, 40, 41), but their roles and influences on viral replication remain undescribed or at least controversial. p53 also localizes to sites of viral DNA replication (42) and positively regulates HSV-1 replication (43) and the progression to encephalitis (44). However, the mechanism of action has not been elucidated.

In this study, we examined and compared the DDR kinase pathways activated in normal human fibroblasts by input viral DNA, replicating/progeny viral DNA, and etoposide treatment. We observed distinct DDR kinase responses for uninfected versus infected cells, including a biphasic DDR to infection dominated by an initial Chk2 response to incoming viral DNA, which then transitioned to an ATM-dominated response to replicating viral DNA. Moreover, we observed that ATM, but not Chk2, promoted efficient viral replication. ATM was also responsible for the phosphorylation of all of the DDR proteins tested, further solidifying ATM as a dominant kinase during HSV-1 infection. Interestingly, we report Mre11-dependent restriction of replication, indicating that Mre11 and ATM may play independent, and possibly opposing, roles during infection. Finally, we observed that p53 was phosphorylated by ATM and promoted viral transcription of the essential viral *ICP8* gene. Our results uncover novel roles for the DDR in regulating HSV-1 gene expression and overall replication.

## RESULTS

### Distinct DNA damage response kinase activation to input and replicating viral DNA.

**FIG 1:**
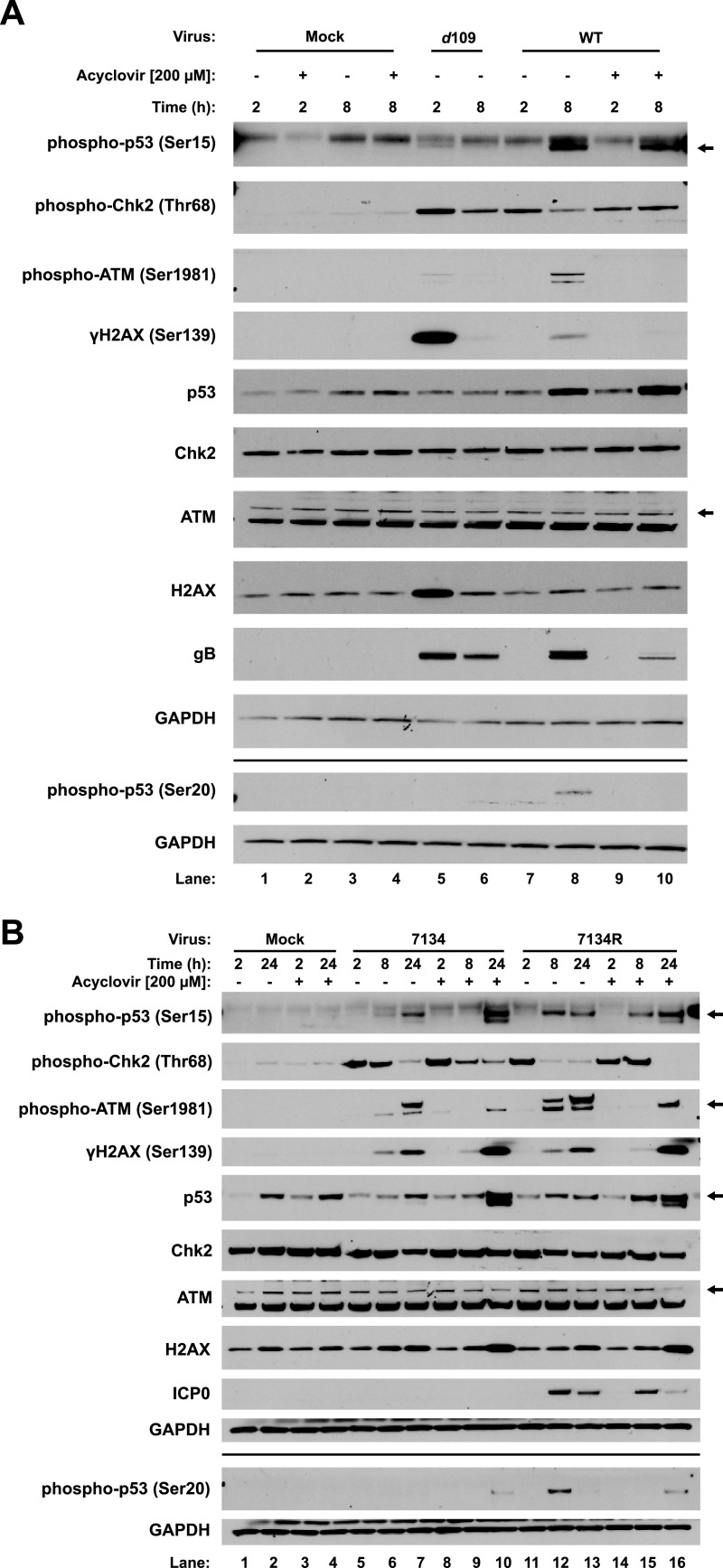
Biphasic DDR kinase response to incoming and replicating viral DNA. Primary HFFs were either mock infected or infected with HSV-1 *d*109 or WT virus at a multiplicity of infection (MOI) of 5 in either the presence or absence of 200 μM ACV added 1 hpi in the case of WT virus. Lysates were harvested for immunoblot analysis at 2 and 8 hpi (A). HFFs were also infected with 7134 or 7134R virus at an MOI of 5 with and without 200 μM ACV treatment 1 hpi, and lysates were harvested at 2, 8, and 24 hpi (B). Immunoblots presented are representative of two independent experiments. “Mock” indicates infection medium alone (no virus).

The life cycle of HSV-1 can be divided into two stages: pre- and post-viral DNA replication. To characterize the response to input viral DNA, we infected primary human foreskin fibroblasts (HFFs) with HSV-1 *d*109. This virus expresses low levels of all genes in normal cells, and nuclear viral DNA persists in a quiescent state for at least 28 days in cell culture (45), so it is a good model for the study of input viral DNA delivered by infection. We investigated the responses at 2 and 8 h postinfection, the times of the onset of initial viral gene expression and of viral genome replication and robust gene expression, respectively (46). By 2 h after *d*109 virus infection, we observed robust Chk2 and H2AX phosphorylation, along with a substantial upregulation of total H2AX, low levels of p53 phosphorylation at serine 15, and low levels of ATM phosphorylation (Fig. 1A, lane 5). This argued that Chk2 was activated by incoming viral DNA. Upregulation of H2AX in response to an activated DDR has been described previously (47), and our results argued that *d*109 infection was sufficient to trigger this response. By 8 h postinfection (hpi), phosphorylation of these proteins was decreased (Fig. 1A, lane 6), presumably due to resolution of the DDR.

To investigate the responses to replicating and replicated viral DNA, we infected HFFs with HSV-1 wild type (WT) virus in either the presence or absence of the viral DNA replication inhibitor acyclovir (ACV). In infected cells undergoing viral DNA synthesis (i.e., without ACV), we observed strong Chk2 phosphorylation by 2 hpi (Fig. 1A, lane 7), like *d*109 infection, that decreased by 8 hpi (lane 8). Interestingly, treatment with ACV to block viral DNA synthesis abrogated this reduction (Fig. 1A, lanes 9 and 10), arguing that viral DNA replication or a viral late gene product suppressed Chk2. We observed ATM, H2AX, and p53 serine 15 and 20 phosphorylation by 8 hpi (Fig. 1A, lane 8), and all but serine 15 phosphorylation were sensitive to ACV treatment (Fig. 1A, lane 10), arguing that viral DNA replication increased levels of phosphorylated forms of these proteins. The phosphorylation of serine 15 with ACV treatment (lane 10) suggested that phosphorylation occurred between 2 hpi and the onset of genome replication, perhaps by the prolonged presence of the parental genome or IE and E gene expression. We did not observe H2AX phosphorylation at 2 hpi following WT infection (Fig. 1A, lane 7), as we did with *d*109. We believe that while *d*109 is an accurate model for input viral DNA, the virus prep of *d*109 likely contained a higher number of viral particles per PFU than the WT virus prep, evidenced by the abundance of gB glycoproteins (Fig. 1A, lanes 5 and 7). This could have led to a stronger H2AX response than WT virus infection as a result of a higher abundance of input genomes when we used the same multiplicity of infection (MOI). Together, these observations indicated a biphasic DDR to input and replicating viral DNA, with Chk2 being activated primarily by incoming viral genomes and ATM being activated by replicating/progeny DNA.

### Effect of the HSV-1 ICP0 protein on the DDR.

Prior studies had observed that the viral ICP0 E3 ubiquitin ligase can promote ATM and Chk2 phosphorylation (48), inhibit ATRIP/ATR function (39), and inhibit downstream repair processes by promoting the degradation of the RNF8 and RNF168 E3 ubiquitin ligases (33). To investigate the effects of ICP0 in our experimental system, we infected HFFs with the ICP0-null 7134 virus and the repaired 7134R virus (49). Similar to WT virus, Chk2 was phosphorylated by 2 hpi (Fig. 1B, lanes 5 and 11), independently of ICP0, and decreased over the course of infection (Fig. 1B, lanes 6 and 12). The decrease was not as rapid with 7134 infection as with 7134R, and we attributed this to the overall slower replication kinetics of the virus without ICP0. We observed ATM phosphorylation by 8 hpi with both 7134 and 7134R (Fig. 1B, lanes 6 and 12), with the 8 h time point being sensitive to ACV treatment (lanes 9 and 15). Interestingly, by 24 hpi in the presence of ACV, ATM was phosphorylated for both viruses (Fig. 1B, lanes 10 and 16), suggesting that, despite replication being inhibited, prolonged presence of the viral genome and/or prolonged expression of viral IE and E genes led to ATM activation. H2AX phosphorylation followed a similar profile (Fig. 1B, lanes 10 and 16). H2AX phosphorylation was reduced at 8 hpi with ACV treatment for both viruses (Fig. 1B, lanes 9 and 15) but was independent of viral DNA replication and viral late gene expression by 24 hpi (Fig. 1B, lanes 10 and 16). Together, our results argued that the DDR kinase responses to 7134 and 7134R were largely similar, with minor differences attributed to the slower replication kinetics of the ICP0^−^ virus.

We also observed phosphorylation of p53 serine 15 with both viruses (Fig. 1B, lanes 7 and 13). However, phosphorylation in the absence of ICP0 occurred much more slowly, occurring primarily at 24 hpi. We also attributed this, as with Chk2 phosphorylation, to the decreased replication kinetics of the 7134 virus. Similar to WT infection, p53 serine 15 phosphorylation was not sensitive to ACV treatment for both viruses (Fig. 1B, lanes 10 and 16), arguing that phosphorylation of this residue was independent of viral DNA replication and L gene expression. Interestingly, we observed very little phosphorylation of p53 on serine 20 following infection with 7134 (Fig. 1B, lanes 5 to 10). Because we observed that phosphorylation of this residue was dependent on viral DNA replication following WT infection, we hypothesized that 7134 did not undergo sufficient levels of DNA replication to stimulate phosphorylation. In agreement with WT infection, 7134R infection stimulated phosphorylation of p53 serine 20 by 8 hpi (Fig. 1B, lane 12) that was reduced by ACV treatment (lane 15). Interestingly, we observed serine 20 phosphorylation of p53 in 7134 virus-infected cells by 24 hpi in the presence of ACV (Fig. 1B, lane 10), arguing that, as in the case of ATM phosphorylation, prolonged presence of viral DNA and/or viral gene expression can stimulate p53 phosphorylation.

Thus, we did not observe any significant qualitative differences between ICP0-null and ICP0-expressing viruses. We observed only minor differences in the kinetics of phosphorylation of DDR proteins, most likely due to the decreased replication kinetics of the ICP0-null 7134 virus.

### ATM promotes the DDR to viral infection.

**FIG 2:**
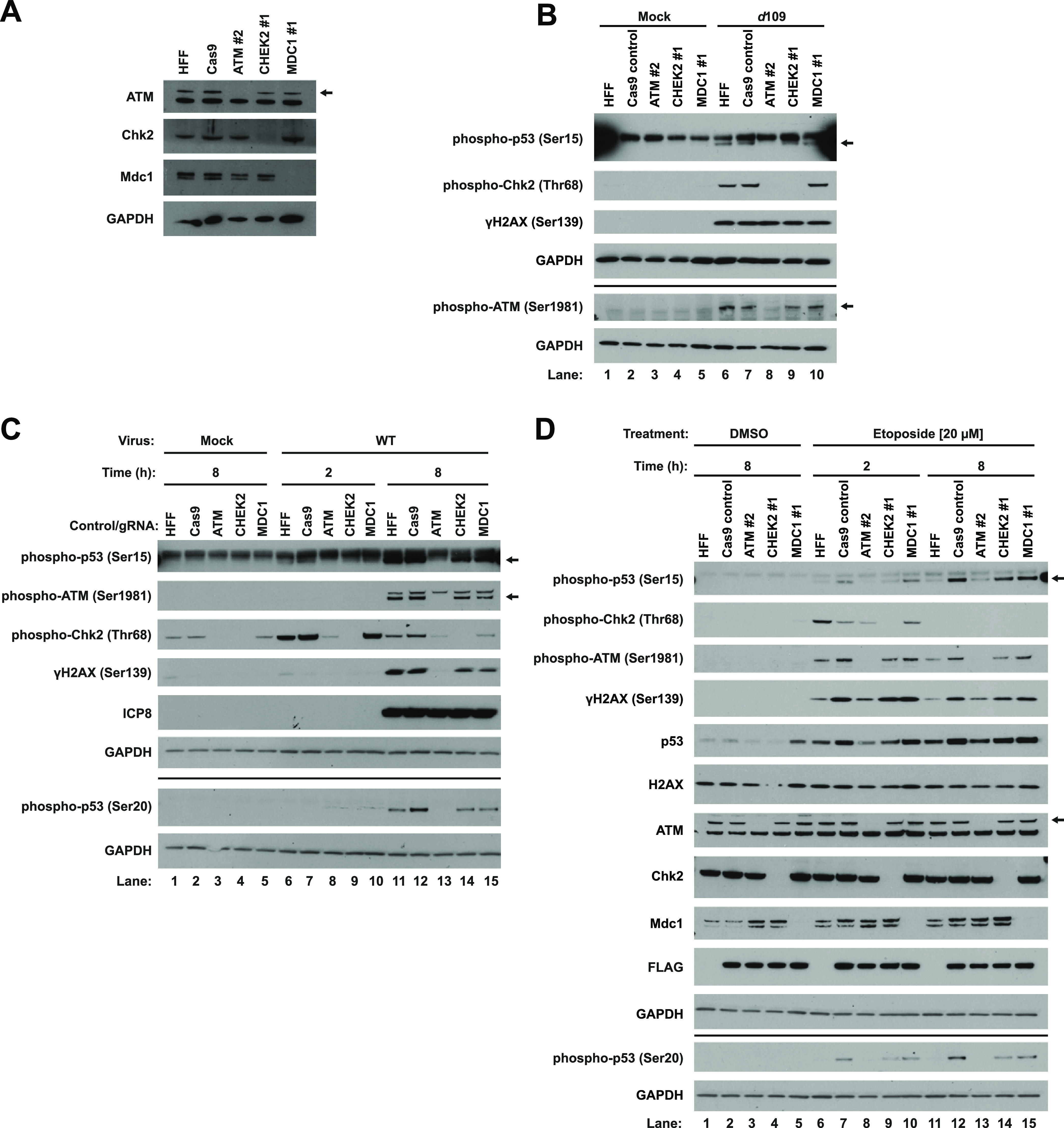
ATM promotes the DDR to both cellular DNA damage and HSV-1 infection. Primary HFFs were transduced with lentiviruses that express Cas9, a guide RNA against ATM, CHEK2, or MDC1, and puromycin resistance as specified in Materials and Methods. Blot images are representative of three passages of cells (A). Normal HFFs, Cas9 control, and knockout HFFs were mock infected or infected with *d*109 at an MOI of 5, and lysates were harvested for immunoblotting at 2 hpi (B). Control and knockout cells were either mock infected or infected with WT virus at an MOI of 5, and lysates were harvested at the indicated times (C). Control and knockout HFFs were treated with either DMSO or 20 μM etoposide, and lysates were harvested at the indicated times posttreatment (D). Cas9 was FLAG tagged. Blots are representative of two independent experiments (B to D).

Having characterized the kinase pathway responses to input and replicating viral DNA, we further sought to determine the kinase(s) responsible for p53, H2AX, ATM, and Chk2 phosphorylation and whether the kinase(s) responsible is different between input and replicating DNA. We generated gene knockouts of ATM and CHEK2 in primary HFFs using clustered regularly interspaced palindromic repeats (CRISPR)-Cas9 (Fig. 2A). We also generated a knockout cell line for MDC1. MDC1 has been observed to localize adjacent to incoming viral genomes (33), but its role during HSV-1 replication has not been investigated. To determine the kinases responsible for the response to input DNA, we infected the knockout HFFs with *d*109 for 2 h. Knockout of ATM ablated Chk2 and p53 serine 15 phosphorylation (Fig. 2B, lane 8) compared to both normal and Cas9 control HFFs (Fig. 2B, lanes 6 and 7), arguing that ATM was required for the phosphorylation of both of these proteins. Knockout of MDC1 had no effect on the phosphorylation of any of the proteins investigated (Fig. 2B, line 10), suggesting that Mdc1 did not have a role in promoting the DDR in the first 2 hpi. Surprisingly, knockout of ATM had no effect on H2AX phosphorylation (Fig. 2B, lane 8). DNA-PK also phosphorylates H2AX in response to DNA damage (50, 51), and we hypothesized that this kinase may be responsible for the H2AX phosphorylation response to input DNA. To test this, we knocked down *PRKDC*, encoding DNA-PKcs, and infected the knockdown cells with *d*109 virus for 2 h. Compared to nontargeting small interfering RNA (siRNA), γH2AX was only partially reduced with knockdown (see Fig. S1A, lanes 3 and 4, in the supplemental material), arguing that DNA-PK functioned redundantly with another kinase. HSV-1 infection also activates the ATR kinase known to also phosphorylate H2AX (38, 52). Therefore, DNA-PK may function redundantly with ATR and ATM.

10.1128/mBio.03552-20.1 FIG S1 Primary HFFs were transfected with either a nontargeting siRNA pool or a pool targeting DNA-PKcs (the gene product of *PRKDC*). Three days later, cells were infected with *d*109 at an MOI of 5, and protein lysates were collected for immunoblotting 2 h later (A). Plain HFFs, Cas9 control, ATM #2, CHEK2 #1, and MDC1 #1 knockout cells were either mock infected, infected with 7134 (B), or infected with 7134R (C), both at an MOI of 5, and protein lysates were harvested at the indicated times postinfection. Blots are representative of two independent experiments. Download FIG S1, PDF file, 0.2 MB. Copyright © 2021 Mertens and Knipe. 2021 Mertens and Knipe. This content is distributed under the terms of the Creative Commons Attribution 4.0 International license.

To determine if ATM, Chk2, and Mdc1 play any role following viral DNA replication, we infected the control and knockout HFFs with WT virus. By 2 hpi, the results mirrored *d*109 infection, where knockout of ATM strongly reduced the phosphorylation of Chk2 (Fig. 2C, lane 8). By 8 hpi, knockout of ATM ablated the phosphorylation of Chk2 and p53 at both serine residues (Fig. 2C, lane 13), arguing that ATM was largely responsible for progression of the DDR in response to DNA replication. Interestingly, knockout of Chk2 had no effect on the phosphorylation of p53 on serine 20 (Fig. 2C, lane 14). Because a previous publication reported that Chk2 promoted p53 phosphorylation in response to cellular DNA damage (16), this may be an HSV-specific effect. In addition, ATM knockout abolished H2AX phosphorylation by 8 hpi (Fig. 2C, lane 13), arguing that ATM phosphorylated H2AX stimulated by replicating viral DNA, not incoming DNA. Knockout of MDC1 had no effect on any of the proteins investigated (Fig. 2C, lanes 10 and 15), indicating a dispensable role for Mdc1 in propagating the DDR.

Having established the effects on wild-type virus, we wanted to determine whether similar results would be observed in the absence of ICP0. To test this, we infected control and knockout HFFs with 7134 and 7134R. For both viruses, ATM was still responsible for the phosphorylation of Chk2, H2AX, and both serine residues of p53 (Fig. S1B and C, lanes 12 to 14), arguing that the ATM dependency on propagating the DNA damage signal was independent of ICP0.

### Etoposide induces a robust DDR in HFFs, distinct from HSV-1 infection.

To investigate whether the response to cellular DNA damage was similar to or different from the response to viral infection, we treated control and knockout HFFs with etoposide to induce double-stranded DNA breaks for 2 and 8 h. By 2 h, we observed phosphorylation of ATM, Chk2, H2AX, and p53 on both serine residues (Fig. 2D, lanes 6 and 7). Interestingly, unlike HSV-1 infection, Chk2 phosphorylation was minimally dependent on ATM, as ATM knockout had only a small effect on Chk2 phosphorylation compared to Cas9 control cells (Fig. 2D, lane 8). γH2AX accumulation was also only partially dependent on ATM (Fig. 2D, lanes 8 and 13), indicating that, as with *d*109 infection, ATM may function redundantly with other DDR kinases to phosphorylate this histone variant. p53 phosphorylation on serines 15 and 20 was also dependent on ATM. Interestingly, we observed serine 20 phosphorylation at 2 h (Fig. 2D, lane 7), whereas for HSV-1, it was not observed until later times during infection, primarily stimulated by viral DNA replication. ATM, H2AX, and p53 phosphorylation persisted through 8 h posttreatment (Fig. 2D, lanes 11 and 12). Chk2 phosphorylation mirrored what was observed for HSV-1 infection. Its phosphorylation was observed beginning at 2 h (Fig. 2D, lanes 6 and 7) but was reduced by 8 h (lane 11 and 12). We attributed this to a resolution in the Chk2 arm of the DDR. These observations also argued that this Chk2 arm of the response was independent of ATM activity as initial Chk2 phosphorylation was not dependent on ATM, and Chk2 phosphorylation decreased over time whereas ATM phosphorylation levels remained constant (Fig. 2D, lanes 6, 7, 11, and 12). Phosphorylation of both p53 serine residues remained dependent on ATM at 8 h posttreatment (Fig. 2D, lane 13).

Together, our results argued that the cellular DNA damage response, at least with respect to damage caused by etoposide, was distinct from the response to HSV-1 infection. Etoposide treatment led to robust phosphorylation of all of the proteins investigated by 2 h, whereas for HSV-1 infection, protein phosphorylation was temporally separated, with the bulk of Chk2 and ATM phosphorylation occurring by 2 and 8 h postinfection, respectively. Chk2 phosphorylation also displayed various requirements for ATM depending on whether the response was triggered by cellular DNA damage or viral infection, indicating that viral gene products were manipulating the host DDR or that cellular and viral DNA were treated differently by cellular surveillance pathways.

### Mre11 promotes distinct DDRs to cellular DNA damage and HSV-1 infection.

**FIG 3:**
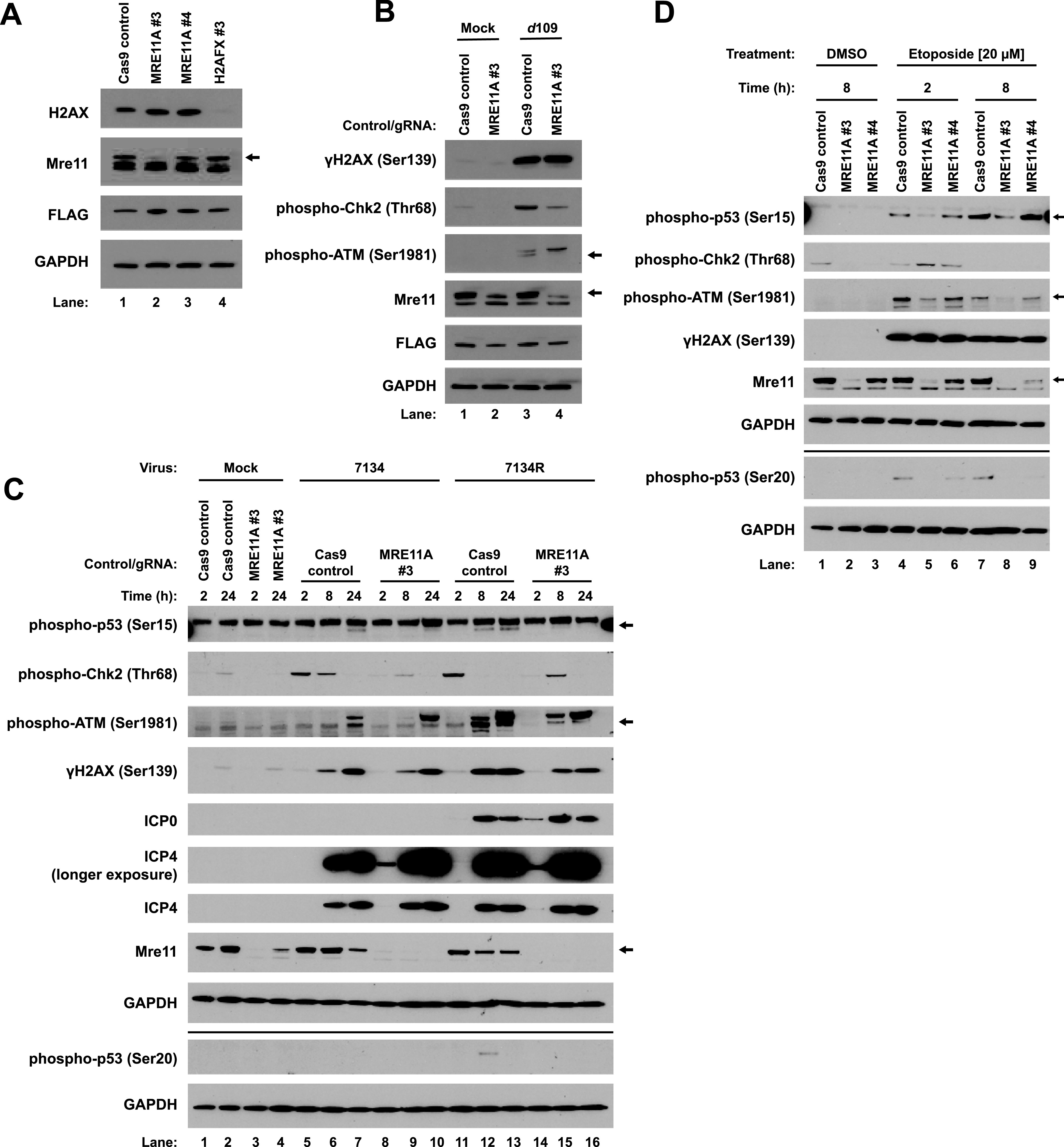
Mre11 influences the response to incoming and replicating viral DNA. Primary HFFs were transduced with lentiviruses that express Cas9 only or Cas9 along with one of two guide RNAs against MRE11A or a guide RNA against H2AFX, as outlined in Materials and Methods. Blots are representative of three passages of cells (A). Cas9 control and MRE11A #3 cells were either mock infected or infected with *d*109 at an MOI of 5, and lysates were harvested at 2 hpi (B). Cas9 control and MRE11A #3 cells were mock infected or infected with 7134 or 7134R at an MOI of 5 and lysates harvested at 2, 8, and 24 hpi (C). Cas9, MRE11A #3, and MRE11A #4 cells were treated with either DMSO or 20 μM etoposide, and lysates were harvested at the indicated times posttreatment (D). Blots are representative of two independent experiments (B to D).

Having observed the requirement of ATM for a robust DDR, we hypothesized that the MRN complex may have facilitated this phenotype by activating ATM in response to infection. Additionally, we sought to determine whether the H2AX histone variant has a role in regulating replication. One study reported that γH2AX has no impact on replication (36), but another study reported that H2AX enhanced replication (33). To test the roles of these proteins, we generated knockout HFFs for the genes MRE11A and H2AFX, encoding Mre11 and H2AX, respectively. MRE11A 3 (Fig. 3A, lane 2) gave a robust knockout, whereas MRE11A 4 (lane 3) gave an intermediate knockout, between those of Cas9 control and MRE11A 3 cells. H2AX levels were not detectable in H2AFX 3 cells (Fig. 3A, lane 4).

First, to determine whether the MRN complex facilitates the DDR to input viral DNA, we infected control and MRE11A 3 cells with *d*109 for 2 h. Loss of Mre11 reduced both ATM and Chk2, but not H2AX, phosphorylation (Fig. 3B, lane 4). This agreed with our ATM knockout results, where H2AX phosphorylation was also unaffected (Fig. 2B). Our results argued that the MRN complex was responsible for inducing an H2AX-independent DDR to incoming viral DNA.

Second, to determine whether the MRN complex promotes the DDR to replicating viral DNA, and also whether ICP0 has any role in this response, we infected control and MRE11A 3 knockout HFFs with the 7134 and 7134R viruses. Mre11 promoted Chk2 phosphorylation in response to both viruses by 2 h (Fig. 3C, lanes 8 and 14), indicating that, as with *d*109 infection, Mre11 and presumably the MRN complex were required for early recognition of incoming viral DNA. By 8 hpi with both 7134 and 7134R infection, ATM and H2AX phosphorylation were only weakly affected by MRE11A knockout (Fig. 3C, lanes 9 and 15), arguing that there were redundant pathways for their activation. p53 serine 15 phosphorylation was strongly reduced by 8 hpi with both viruses with MRE11A knockout (Fig. 3C, lanes 9 and 15), arguing that Mre11 primarily facilitated phosphorylation of this residue, most likely through ATM. p53 serine 20 phosphorylation following 7134R infection was also sensitive to Mre11 depletion at 8 hpi (Fig. 3C, lane 15), also arguing that Mre11 controlled phosphorylation of this residue of p53 as well. We also observed increased ICP4 and ICP0 protein levels with MRE11A knockout by 2 hpi for both ICP0^−^ and ICP0^+^ viruses (Fig. 3C, lanes 8 and 14), compared to the Cas9 control (lanes 5 and 11), providing evidence for a role for Mre11 in repression of IE gene expression.

To determine if Mre11 promoted a similar DDR to cellular damage, we treated control and MRE11A #3 and #4 cells with etoposide for 2 and 8 h. The results were very similar to those observed for ATM knockout. MRE11 #3 cells displayed reduced ATM and p53 serine 15 and 20 phosphorylation at both 2 and 8 h posttreatment (Fig. 3D, lanes 5 and 8). In agreement with our ATM knockout results, Chk2 phosphorylation was not reduced by the loss of Mre11 (Fig. 3D, lane 5), in fact Chk2 phosphorylation appeared to be increased relative to the Cas9 control cell line, arguing that Mre11 repressed Chk2 activation with etoposide treatment but promoted its activation following HSV-1 infection. γH2AX accumulation appeared to be independent of Mre11 (Fig. 3D, lane 5), again arguing for a redundancy in the kinases responsible, similar to our observations for HSV-1 infection. MRE11 #4 gave phenotypes similar to those seen in MRE11A #3 cells (Fig. 3D, lanes 6 and 9), but not as drastic. We attributed this to the incomplete knockout of MRE11A compared to MRE11A 3 cells. Together, our results indicated that Mre11 promoted the activation of ATM following both etoposide treatment and HSV-1 infection but were divergent with regard to Chk2 phosphorylation; ATM promoted its phosphorylation following HSV-1 infection but not etoposide treatment. p53 phosphorylation was promoted by both Mre11 and ATM, indicating that this arm of the DDR pathway was conserved between chemical treatment and infection.

### ATM and Mre11 have opposing effects on HSV-1 replication.

**FIG 4:**
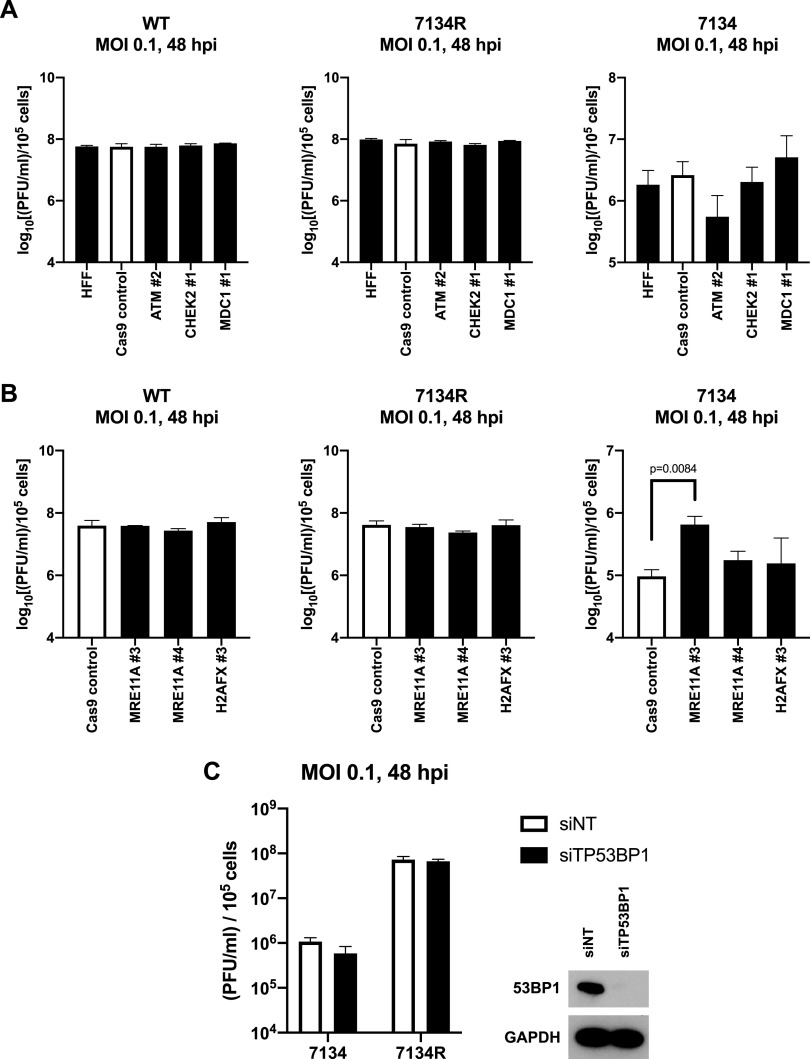
ATM and Mre11 have different roles in regulating the replication of HSV-1. Normal HFFs, Cas9 control, ATM 2, CHEK2 1, and MDC1 1 knockout cells were infected with WT, 7134R, and 7134 viruses at an MOI of 0.1, and total virus was collected 48 h later and titrated on U2OS cells (A). Cas9, MRE11A #3, MRE11A #4, and H2AFX #3 were also infected with WT, 7134R, and 7134 viruses at an MOI of 0.1 for 48 h, and total virus was quantified via plaque assay on U2OS cells (B). Data are expressed as the log_10_ values of the plaque counts normalized to the number of initially infected knockout cells (A and B). Primary HFFs were transfected with either a nontargeting siRNA pool or a pool targeting TP53BP1. Knockdown cells were infected with 7134 and 7134R at an MOI of 0.1 for 48 h, and total virus was quantified by plaque assays on U2OS cells. Protein lysates for knockdown verification were taken at the time of infection (C). Data are averages from three independent experiments, with error bars representing the standard errors of the means (SEM). Statistical analysis was performed using an unpaired *t* test.

Prior studies have reported conflicting results as to whether various DDR proteins regulate the replication of HSV-1. To address these differences, we infected our ATM, CHEK2, and MDC1 knockout HFFs with WT, 7134R, and 7134 viruses and measured viral yields by plaque assay. Replication of both WT and 7134R viruses was unaffected by all of the gene knockouts (Fig. 4A). However, knockout of ATM demonstrated a trend toward reduction of replication of the 7134 virus, indicating that ATM had proviral functions, but only in the absence of ICP0. Interestingly, knockout of CHEK2 did not have an effect on any of the viruses. A prior study observed that loss of Chk2 decreased viral replication, but only in the presence of ICP0 (48). Our results argued that although ATM promoted the activation of Chk2, the latter was ultimately dispensable for HSV-1 replication, independent of ICP0 expression. Similarly, knockout of MDC1 affected the replication of the 7134 virus (Fig. 4A) only marginally.

Having observed that DNA-PKcs partially promoted H2AX phosphorylation in response to incoming viral genomes, we next sought to determine its effect on replication. DNA-PKcs is known to be a target of ICP0 and is degraded during infection (53). However, we previously observed coprecipitation of DNA-PKcs with the viral DNA replication protein ICP8 during infection with wild-type virus, suggesting that degradation may not occur in all cell types (34). To test this in our primary fibroblast model system, we infected HFFs with 7134 and 7134R. Over time, DNA-PKcs was degraded with 7134R, but not 7134, infection, indicating that DNA-PKcs is a target of ICP0 in HFFs (Fig. S2A). Depletion of DNA-PKcs had no effect on the replication of 7134 and 7134R (Fig. S2B), indicating that DNA-PKcs did not regulate replication in primary fibroblasts. This was surprising, as a previous study observed that human gliomal cells deficient in DNA-PK function supported higher levels of viral replication than a control cell line (54). Additionally, we observed previously that murine cells lacking Ku70, a protein necessary for DNA-PKcs localization, also exhibited enhanced replication of the virus (34). Our results may argue that the different subunits of the DNA-PK complex may have different roles in the HSV-1 life cycle in different cell types, the extent of which remains to be determined.

10.1128/mBio.03552-20.2 FIG S2 HFFs were either mock infected or infected with 7134 or 7134R at an MOI of 5, and protein lysates were collected at the indicated times postinfection (A). Blot images are representative of two independent experiments. Primary HFFs were transfected with either a nontargeting siRNA or DNA-PKcs-specific siRNA pools for three days. Cells were then infected with either 7134 or 7134R at an MOI of 0.1, and total virus was collected after 48 h and quantified via plaque assay on U2OS cells (B). Data shown are the averages with SEM of three independent experiments. Download FIG S2, PDF file, 0.2 MB. Copyright © 2021 Mertens and Knipe. 2021 Mertens and Knipe. This content is distributed under the terms of the Creative Commons Attribution 4.0 International license.

To investigate the effects of Mre11 and H2AX loss on replication, we infected our knockout HFFs with WT, 7134R, and 7134 viruses and quantified progeny virus by plaque assay. Surprisingly, loss of Mre11 significantly enhanced replication of 7134 and did not affect 7134R or KOS (Fig. 4B), arguing that Mre11 restricted HSV-1 replication in the absence of ICP0. Mre11 has been reported to be lost over the course of infection in a manner independent of ICP0 (37). We observed a slight reduction in Mre11 levels at later times during 7134 but not 7134R infection (Fig. 3C), indicating that ICP0 may target some other protein in the restriction pathway of which Mre11 is a component. Loss of H2AX had no effect on replication of the three viruses (Fig. 4B), consistent with a previous report (36).

In the absence of ICP0, the downstream DDR protein 53BP1 localizes to incoming viral genomes (33). However, it is unknown whether this has any role in the regulation of viral replication. To test this, we depleted 53BP1 in primary HFFs using siRNAs and measured the replication of 7134 and 7134R by plaque assay. Despite successful knockdown, replication of both viruses was not affected (Fig. 4C), arguing that 53BP1 was dispensable for replication. Recruitment of 53BP1 to viral genomes is dependent on H2AX (33). Our results thus far have argued that this H2AX arm of the ATM pathway did not impact the replication cycle of HSV-1 and the actions of ATM were through some other mechanism. Our observations also revealed a novel function of Mre11 in primary fibroblasts for restriction of replication of an ICP0^−^ virus. Furthermore, these contrasting phenotypes between ATM and Mre11 suggested that while Mre11 promoted the partial activation of ATM, these proteins had separate functions in regulating the replication of the virus outside their roles in propagating the DDR.

### p53 promotes the replication of ICP0-null HSV-1.

**FIG 5:**
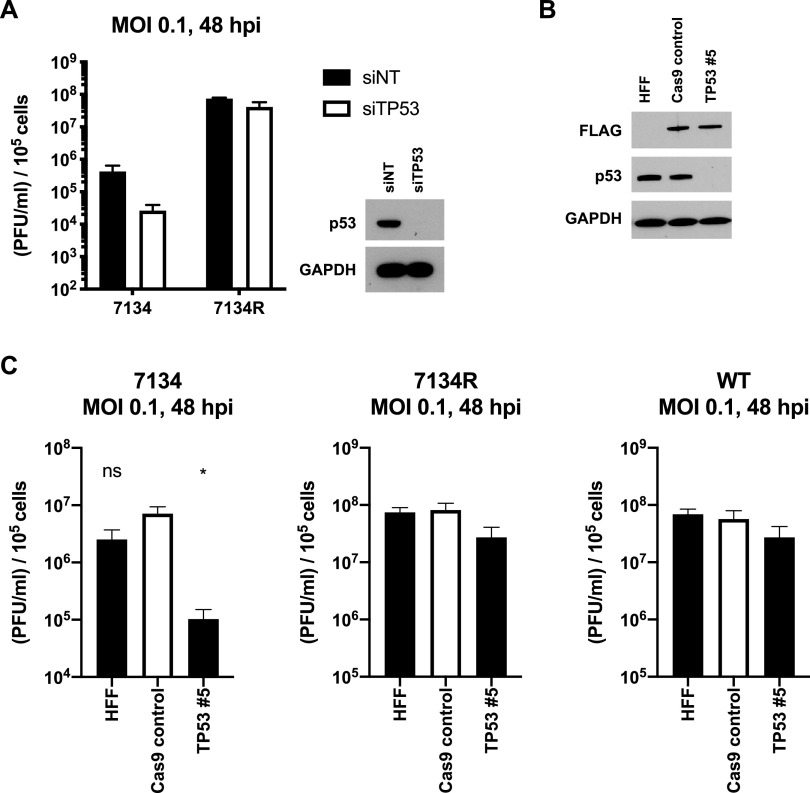
p53 positively regulates the replication of ICP0-null HSV-1. Primary HFFs were transfected with a nontargeting siRNA pool or a pool targeting TP53 and infected with 7134 and 7134R 3 days later at an MOI of 0.1 for 48 h, and total virus was quantified on U2OS cells via plaque assay. Protein lysates were also collected at the time of infection to verify the knockdown (A). Primary HFFs were transduced with lentiviruses expressing Cas9 only or Cas9 along with a guide RNA against TP53. The extent of the knockout was verified via immunoblotting after roughly 2 weeks of puromycin selection (B). Blots are representative of three passages of cells. Normal HFFs, Cas9 control, and TP53 5 cells were infected with 7134, 7134R, and WT viruses at an MOI of 0.1 for 48 h, and total virus was quantified via plaque assay on U2OS cells (C). Graphed data are the averages of three independent experiments (A and C). Statistical significance was determined, compared to the Cas9 control cell line, using unpaired *t* tests. *, *P* < 0.05; ns, not significant. Errors bars represent the SEM.

ATM is required for p53 phosphorylation on serines 15 and 20 (55), and our observations were consistent with that study. Despite its being known to promote replication, it is unknown precisely where p53 acts in the viral replication cycle. To determine whether p53 regulates replication in primary HFFs, we knocked it down with siRNA and measured the replication of 7134 and 7134R. Knockdown of p53 reduced replication of 7134 by roughly 10-fold (Fig. 5A), but 7134R replication was unaffected. These findings indicated that p53 promoted replication of an ICP0-null virus in primary fibroblasts. Next, using CRISPR-Cas9, we generated a gene knockout of TP53 in HFFs (Fig. 5B). In agreement with our siRNA results, knockout of TP53 significantly reduced the replication of 7134 but not 7134R. Furthermore, WT virus was also unaffected by the knockout (Fig. 5C). Together, our results argued that p53 is proviral, but its absence can be compensated for by ICP0. In contrast, we observed that an increased abundance of p53 did not enhance replication. We treated HFFs with increasing concentrations of nutlin-3a to increase the levels of p53 (56), and despite heightened levels of p53 (Fig. S3A), replication of all three viruses was not affected (Fig. S3B). This indicated that only a loss, and not an overabundance, of p53 has an effect on replication, indicating that p53 was required but not limiting for HSV-1 infection.

10.1128/mBio.03552-20.3 FIG S3 HFFs were either untreated or treated with the indicated concentration of nutlin-3a, and protein lysates were prepared at the indicated times posttreatment. Blots are representative of three independent experiments (A). HFFs were pretreated with the indicated amount of nutlin-3a for 1 h and then infected with 7134, 7134R, or WT viruses at an MOI of 0.1. Cells received nutlin-3a treatment throughout the course of infection. Total virus was harvested at 48 hpi and quantified via plaque assay on U2OS cells (B). For the DMSO-only control, the volume of DMSO was the same as that of nutlin-3a in the 10 μM nutlin-3a condition. Data shown are averages of three independent experiments with SEM. Download FIG S3, PDF file, 0.2 MB. Copyright © 2021 Mertens and Knipe. 2021 Mertens and Knipe. This content is distributed under the terms of the Creative Commons Attribution 4.0 International license.

### p53 promotes the transcription of an essential viral gene encoding a DNA replication protein.

**FIG 6:**
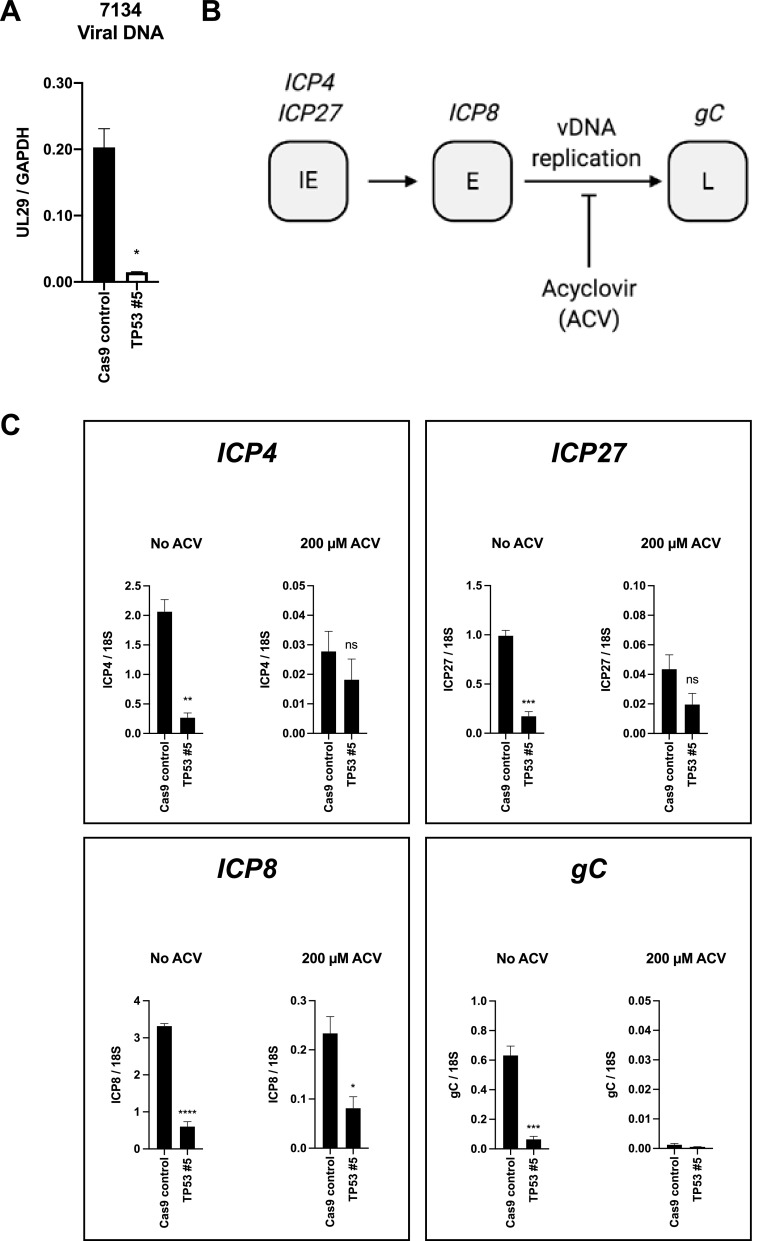
p53 promotes prereplication *ICP8*, but not *ICP4* or *ICP27*, gene transcription. Cas9 and TP53 #5 cells were infected with 7134 at an MOI of 1, and nucleic acids were collected 24 h later. Purified DNA was amplified and quantified via real-time PCR with primer pairs for the *UL29* locus of the viral genome and cellular *GAPDH*. The values for *UL29* were normalized to the *GAPDH* values (A). cDNAs of viral genes representative of each viral gene class were quantified. *ICP4* and *ICP27* represented the IE class, *ICP8* the E class, and *g*C the L class. Cells were treated with ACV to allow the quantification of IE and E transcripts before the onset of viral DNA replication (B). Total RNA from the same samples collected for panel A was isolated and reverse transcribed, and the indicated viral transcripts were quantified via real-time PCR and normalized to cellular 18S rRNA transcripts (C). Data are averages with SEM from three independent experiments (A and C). Statistical significance was determined, compared to the Cas9 control cells, via unpaired *t* tests. *, *P* < 0.05; **, *P* < 0.01; ***, *P* < 0.001; ****, *P* < 0.0001; ns, not significant.

Having demonstrated that p53 promoted progeny virus production, we next sought to determine at which step in the virus replication cycle p53 acted. First, we measured viral DNA replication. Knockout of TP53 significantly reduced DNA replication of 7134 (Fig. 6A), while DNA replication of 7134R and WT viruses was marginally affected (Fig. S4A and C, respectively). This indicated that at least part of the defect in progeny virus production in 7134 virus-infected cells was due to reduced replication of the genome. However, before the initiation of DNA replication, HSV-1 must first express the IE genes, which are required to initiate transcription of the E genes, encoding the DNA replication machinery. Therefore, the reduction in viral DNA replication could have been due to a defect in either IE or E gene expression. To determine which gene set was regulated by p53, we treated 7134 virus-infected cells with acyclovir (diagrammed in Fig. 6B). We chose representative genes of each class: *ICP4* and *ICP27* for the IE class, *ICP8* (an essential DNA replication gene) for the E class, and the glycoprotein gene *gC* for the L class. Using acyclovir allowed us to differentiate pre-DNA replication from post-DNA replication gene expression. Without ACV treatment, mRNA levels of the *ICP4*, *ICP27*, *ICP8*, and *gC* genes were reduced significantly in the knockout cells compared to the control line following infection with 7134 (Fig. 6C). This most likely reflected the transcriptional levels of viral mRNA both before and after DNA replication. However, with acyclovir treatment, we observed a significant reduction in *ICP8* gene transcript levels but not *ICP4* and *ICP27* transcripts, arguing that p53 promoted *ICP8*, but not IE, gene transcription, which then led to decreased DNA replication and progeny virus production. In line with our DNA replication results, viral gene transcript levels were comparable between the control and knockout lines with and without acyclovir treatment for 7134R and WT viruses (Fig. S4B and D, respectively). Together, our results indicated that p53 played a pivotal role in promoting the transcription of at least one essential viral DNA replication protein gene, that encoding ICP8.

10.1128/mBio.03552-20.4 FIG S4 Cas9 control and TP53 5 cells were infected with 7134R at an MOI 1, and nucleic acids were collected at 24 hpi. Viral DNA (A) and RNA (B) were quantified using the same protocols as for Fig. 6 via real-time PCR. Control and knockout cells were also infected with WT virus at an MOI of 1 and also harvested at 24 hpi. Viral genomes (C) and RNA (D) were quantified as for panels A and B. Data are averages and SEM from three independent experiments. Download FIG S4, PDF file, 0.1 MB. Copyright © 2021 Mertens and Knipe. 2021 Mertens and Knipe. This content is distributed under the terms of the Creative Commons Attribution 4.0 International license.

IFI16 is a cellular protein known to restrict ICP0-null HSV-1 replication (57). Additionally, IFI16 has been shown to bind to p53 (58, 59) and reported to negatively regulate its function (60), although the latter is still a point of contention (58, 59, 61). To determine whether IFI16 had any role with respect to p53, we knocked down p53 in IFI16 knockout HFFs and measured 7134 yields. Knockdown of p53 resulted in elevated IFI16 protein levels in the Cas9 control cells (Fig. S5A), indicating that p53 negatively regulated the expression of IFI16. Depletion of p53 in both Cas9 control and IFI16 1 cells dramatically reduced 7134 replication compared to nontargeting treatment (Fig. S5B), arguing that IFI16 was not required for p53 function during infection.

10.1128/mBio.03552-20.5 FIG S5 Cas9 control and IFI16 1 knockout cells were transfected with either a nontargeting pool or a pool targeting TP53 for three days. Protein lysates were collected for immunoblotting at the time of the following infections to validate the knockouts and knockdowns (A). Cells were infected with 7134 at an MOI of 0.1. Total virus was collected 48 h later and quantified on U2OS cells via plaque assay (B). Data are the averages of three independent experiments with SEM. Blots are representative of three independent experiments. Download FIG S5, PDF file, 0.1 MB. Copyright © 2021 Mertens and Knipe. 2021 Mertens and Knipe. This content is distributed under the terms of the Creative Commons Attribution 4.0 International license.

## DISCUSSION

Cells infected with HSV-1 are known to activate proteins in the homologous recombination repair pathway (32). The activation starts at early times postinfection and peaks around times of viral DNA synthesis, but the precise effects of input versus replicated viral DNA had not been defined. Furthermore, the DDRs to viral infection have not been compared to normal cell responses to DNA damage agents. In this study, we compared the DDR kinase pathways activated in normal human fibroblasts by input HSV-1 genomic DNA and by HSV-1 replicating/progeny DNA and in uninfected cells treated with etoposide. We also defined the cellular gene products needed for each using CRISPR-Cas9 technology to knock out specific cellular genes. We observed unique DDR kinase pathways for each of these of the situations. We observed that etoposide induced strong phosphorylation of both ATM and Chk2, while input HSV-1 DNA activated a strong Chk2 phosphorylation response, and replicating/progeny HSV-1 DNA activated a strong ATM phosphorylation response. Furthermore, we observed that key DDR proteins acted to regulate HSV-1 infection in that ATM and p53 promoted replication of ICP0-null HSV-1 while Mre11 acted to restrict ICP0-null HSV-1. Individual DDR components can be proviral or antiviral; thus, these results argue that HSV-1 manipulates the host cell DDR to utilize specific components for its optimal replication while inactivating the antiviral aspects of the DDR.

### The DDR to HSV-1 infection is biphasic.

**FIG 7:**
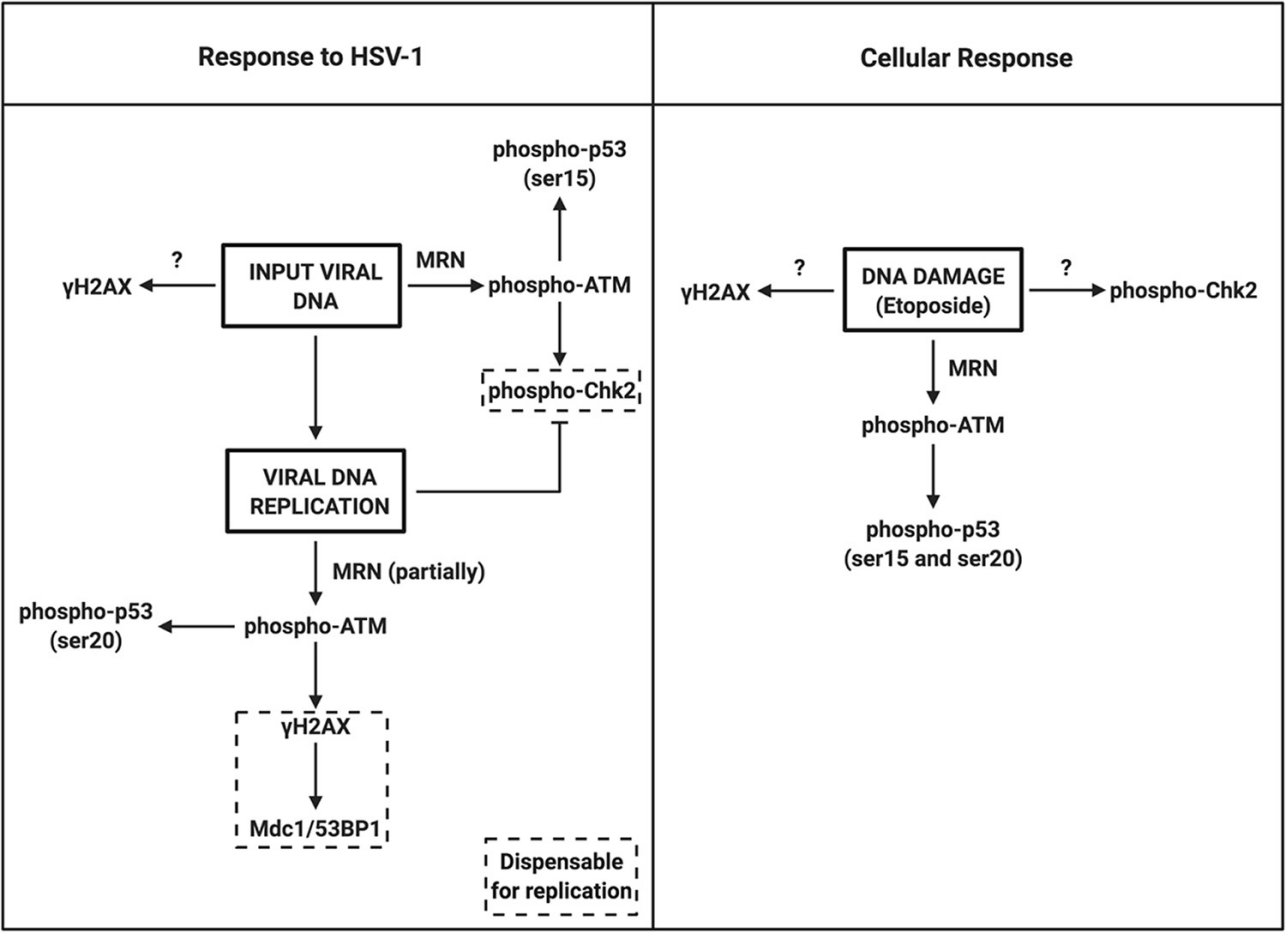
Model for the DDR to HSV-1 infection and the DDR to the cellular genome following etoposide treatment.

HSV-1 lytic infection involves two states of the viral genome, the incoming parental genome and the replicating and replicated progeny genomes. Using mutant viruses and acyclovir to inhibit viral DNA replication, we were able to document the responses to both states and present a model (Fig. 7). The viral genome enters the nucleus as a linear molecule containing not only two free double-stranded ends but also nicks and gaps of various lengths that can activate the DDR (62). We used the *d*109 virus to interrogate the response to the parental genome without any potential effects caused by transcription. We observed that while ATM was responsible for p53 and Chk2 phosphorylation during this phase of the viral replication cycle, γH2AX accumulation was not dependent on ATM. It is conceivable that DNA-PK, ATR, or a redundancy between them and ATM is responsible for γH2AX accumulation. In any case, we observed that H2AX was dispensable for replication, indicating that whichever kinase(s) is responsible, the mechanism by which ATM functions does not involve H2AX. With regard to ATM-dependent Chk2 activation following infection with *d*109, compared to more robust ATM activation (discussed below), this would suggest that ATM specificity may change over the course of infection or perhaps is manipulated by viral gene products. This is exemplified in the transition from ATM-independent to ATM-dependent γH2AX formation during viral genome replication.

Viral genome replication led to further ATM activation, p53 phosphorylation, γH2AX formation, and suppression of Chk2 phosphorylation. At this stage, H2AX phosphorylation was dependent on ATM, indicating that as viral lytic replication progresses, ATM becomes the dominant kinase active, shifting away from Chk2, exhibiting a biphasic modality. Mdc1 and 53BP1 are downstream of ATM, recruited by γH2AX. Interestingly, depletion of these proteins had no effect on replication of the virus, indicating that this arm of the ATM pathway was irrelevant for infection. This may be due, in part, to the relatively small size of the viral genome compared to the cellular genome, as cellular H2AX DDRs span millions of base pairs (63). Histones are loaded onto the viral genome within an hour of initial infection (64). While H2AX associates with the viral genome by 2 h following infection (41) and with newly synthesized genomes (65), γH2AX appears to be excluded from viral replication compartments (39). Therefore, the γH2AX signal that we observed during the viral DNA replication phase may be phosphorylation of H2AX present on the cellular genome by ATM that was activated by viral DNA replication. However, it is possible that HSV-1 replication may induce damage to the cellular genome. Further investigation of ATM substrates is necessary to determine how ATM regulates replication. While Chk2 phosphorylation was ATM-dependent in the context of HSV-1 replication, it was ATM-independent when cells were treated with etoposide. This indicates some specificity in ATM substrates between infection and cellular DNA damage responses. Moreover, we hypothesize that these differences in substrate phosphorylation may be due to active manipulation by the virus to optimize the replication process.

The viral UL12 nuclease binds to the MRN complex and has been proposed, in tandem with ICP8, to facilitate efficient packaging of daughter viral genomes into new capsids (66). One hypothesis is that a UL12/ICP8 complex may localize MRN to viral DNA to activate ATM, at least in part. This manipulation would activate the MRN complex to allow full activation of ATM, which then exerts its proviral role, possibly through p53, while ICP0 inhibits the antiviral effects of Mre11 via degradation of a target protein, or multiple proteins, in the Mre11 pathway. This hypothesis is supported by the fact that we observed replication-dependent ATM activation. Because ICP8 is required for viral DNA replication (67), an ICP8^−^ virus should phenocopy acyclovir treatment. It would be interesting to determine whether viruses lacking UL12 and ICP8 elicit similar DDRs to acyclovir-inhibited WT virus infection.

### Cellular DNA damage and HSV-1 infection are treated differently by the host DDR machinery.

Our observations on the cellular response to DNA damage in the absence of infection are also diagrammed in Fig. 7 as a comparison with HSV-1 infection. We observed that etoposide induces a DDR in HFFs characterized by MRN-dependent ATM activation and p53 phosphorylation. What was interesting, however, was the finding that neither Chk2 nor H2AX phosphorylation is dependent on either Mre11 or ATM, suggesting to us that more than just the canonical MRN-ATM response was activated by etoposide-generated DNA damage. There is precedence for ATM-independent Chk2 phosphorylation (68), although this required prolonged DDR activation, whereas we observed the same phenotype by 2 h. ATR has been shown to phosphorylate Chk2 in response to DNA damage by cisplatin (69), leading us to hypothesize that ATR may have been activated by etoposide treatment, which, in turn, led to the phosphorylation of Chk2. This idea can be extended to H2AX phosphorylation. Because ATR can also phosphorylate this histone variant (52), it is conceivable that this was the underlying mechanism for the apparent ATM-independent phosphorylation of H2AX. DNA-PK is also known to promote the phosphorylation of H2AX (50, 51) and even Chk2 (70), so we may have been observing the effects of all three (ATM, ATR, and DNA-PKcs) kinases simultaneously with etoposide treatment. In addition, HSV-1 does encode two major protein kinases, U_S_3 and U_L_13, that are contained in the virion (71) and expressed as late viral gene products and may phosphorylate DDR proteins. Thus, the different DDRs may be due to effects of viral gene products or differences in the characteristics of the viral and cellular genomes.

Viral DNA is not associated with histones inside the capsid (72). Instead, the viral genome enters as a naked linear molecule that is loaded with histones within 1 h (64, 73). Compared to cellular DNA breaks, the incoming viral genome may not be a sufficient stimulus for full ATM activation, leaving only Chk2 efficiently activated. There is evidence that, as viral DNA replication progresses, the daughter DNA molecules are associated with fewer histone complexes (74). This exposed DNA may be the key feature that leads to ATM activation, and the stimulus from the parental viral genome that was activating Chk2 is no longer present, leading to the observed DNA replication-dependent reduction in Chk2 phosphorylation.

### Mre11 restricts and ATM promotes viral replication.

We observed that Mre11 restricted replication of an ICP0^−^ virus, indicating that ICP0 is responsible for attenuating a different portion of the pathway, as we did not observe changes in Mre11 levels in the presence of ICP0, in contrast to what has been described (37). We also observed that ATM promoted replication of the virus. In the canonical DDR pathway, Mre11 promotes ATM activation, which is in apparent opposition to our findings as Mre11 and ATM depletion had opposing effects on progeny virus production. Mre11 depletion did not totally abolish ATM activation, indicating a redundant mechanism for ATM activation in response to infection. The Tip60 histone acetyltransferase is known to promote ATM activation (75). While it interacts with the MRN complex and its knockdown decreases γH2AX and Rad50 focus formation (76), it remains undetermined whether Tip60 alone is sufficient for ATM activation. This could explain the partial Mre11 independence of ATM activation during HSV-1 infection. The HSV-1 UL13 kinase has been shown to interact with and phosphorylate Tip60 (77). This would provide an elegant mechanism to circumvent Mre11 restriction to promote the proviral functions of ATM; i.e., UL13 phosphorylates Tip60, which in turn acetylates ATM, leading to its phosphorylation and activation. Future experiments will focus on testing this hypothesis. With regard to a mechanism, ATM appears to be the determinant kinase of p53 phosphorylation, and we hypothesize that this phosphorylation is what promotes efficient replication of this virus.

Concerning the restriction phenotype of Mre11, formation of protein complexes known to restrict HSV-1 replication, termed PML (promyelocytic leukemia) nuclear bodies (PML-NBs) or nuclear domain 10 bodies (78), is sensitive to Mre11 depletion. Cells harboring nonfunctional Mre11 accumulated fewer PML-NBs after the cellular genome was damaged by etoposide (79). This could be a potential mechanism of action for Mre11-mediated restriction of ICP0-null HSV-1 replication: association of Mre11 with the viral genome recruits PML-NB proteins to silence viral gene expression and subsequent replication. Our results are consistent with the hypothesis that Mre11 recruits other PML-NB proteins, but ICP0 targets PML-NB proteins for degradation (26) and would therefore attenuate this Mre11 pathway. While our experiments were conducted in primary fibroblasts, we hypothesize that this restriction activity extends to other HSV target cells like keratinocytes, because we observed that normal oral keratinocytes (NOKs) are highly restrictive for ICP0^−^ viruses (80).

Our results also indicate that the mechanism by which Mre11 affects HSV-1 replication may not necessarily be tied to homologous recombination. Viral DNA can undergo HR catalyzed by Rad51/Rad52 (81), and depletion of Rad51 and Rad52 reduced replication when the virus was pretreated with UV radiation (82). Canonically, Mre11 initiates the sensing pathway that leads to HR. Our observations imply a mechanism of Mre11 action that does not involve Rad51/Rad52, as they yield opposing phenotypes. Collectively, our results reveal a novel, or otherwise noncanonical, function of Mre11 outside HR in suppressing the replication of ICP0-null HSV-1.

Others have found that a modified DDR restricts adenovirus replication (22). In that study, both the MRN complex and ATM restricted an adenovirus not expressing E1B-55K/E4-ORF3 through a mechanism where both Mre11 and ATM associated with the viral genome. The MRN complex and ATM also associate with the HSV-1 genome at various points in the replication cycle (40, 41, 65), so it is possible that a similar pathway targets HSV-1. However, in our study, ATM did not restrict HSV-1, indicating that the restriction by Mre11 is not through ATM.

### Role for p53 in the HSV-1 lytic replication cycle.

Previous studies have observed that p53 localizes to sites of viral DNA replication (42) and enhances wild-type viral replication in human colorectal cells (43). In normal HFFs, we observed an effect of p53 on only ICP0^−^ virus, and this may be due to other normal cell functions that are redundant with p53. p53 is ubiquitinated by ICP0 (83), and this modification may serve to inhibit the function of p53 in normal human cells. While this would block any potential apoptosis to prolong progeny virus production, this would come at the expense of its effects on viral gene expression. Because ICP0 is such a potent transactivator of HSV-1 gene expression, this inhibition of p53 may be inconsequential.

The mechanism by which p53 exerts this function is not well understood. We and others (55) have observed that p53 is phosphorylated by ATM during HSV-1 infection. Phosphorylation of serine 15 is crucial for the activity of p53 in regulating gene expression (17). We hypothesize that this may be the mechanism by which ATM exerts its proviral function. p53 binds to the viral genome both *in vitro* and during productive infection, adjacent to the origins of replication (84). The *ICP8* and *ICP4* open reading frames are adjacent to *ori*L and *ori*S, respectively. Therefore, one possible mechanism is that the binding of p53 to the viral genome promotes the transcription of essential viral genes. We observed an effect of p53 on *ICP8* transcription before DNA replication, indicating that p53 function may be restricted to the instances where it binds to *ori*L. The kinetics of association of p53 and the viral genome have not been defined. Time-course genome-wide p53 association studies would help determine not only whether there is a bias for binding to the origins but also whether there are other binding sites not previously described. Coupled with RNA deep sequencing analysis, this would provide a powerful system to investigate where and when p53 binds and whether this also correlates with altered viral gene transcription surrounding these sites.

In summary, our study has revealed novel roles for key proteins in the cellular DNA damage response to HSV infection. Early recognition of damaged DNA by Mre11 had a restrictive effect on HSV-1 replication, while downstream ATM and p53 activation promoted replication. Our observations not only provide new insight into the relationship between the virus and the host but also inform studies of viral reactivation from latency, which is known to be intimately tied to the DDR (85, 86).

## MATERIALS AND METHODS

### Cells, viruses, and infections.

HFF, U2OS, Vero, and HEK293T cells were obtained from the American Type Culture Collection (ATCC) (CRL-1634, HTB-96, CCL-81, and CRL-3216, respectively) and were cultured in Dulbecco’s modified Eagle’s medium (DMEM; Corning) containing 10% (vol/vol) fetal bovine serum (FBS) and penicillin/streptomycin (Pen/Strep). All HSV-1 infections were carried out as described previously (87). Here, DMEM–1% bovine calf serum (BCS) medium is referred to as low-serum medium. The HSV-1 wild-type KOS strain (88) was propagated on Vero cells. The derivative viruses 7134 and 7134R (49) were propagated on U2OS cells. Prior to use, all three viruses were titrated in parallel on U2OS cells. The *d*109 virus (45) was grown on U2OS ICP4/ICP27 cells and titrated on the Vero FO6 cell line as described previously (89).

### Viral yield experiments.

HFF cells (1 × 10^5^ per well in 12-well plates) were infected with the indicated HSV-1 viruses at a multiplicity of infection (MOI) of 0.1 PFU/cell. After 48 h, total virus was collected and titrated by plaque assays as described (57).

### Drug treatments.

The HSV DNA replication inhibitor acyclovir (Sigma) was used to supplement the postinfection low-serum medium to a final concentration of 200 μM. Nutlin-3a (Sigma) was used to supplement low-serum medium to the concentrations indicated in Fig. S3. Control treatments contained only medium (untreated) or a volume of dimethyl sulfoxide (DMSO) equivalent to that of the highest concentration of nutlin-3a used. HFFs were pretreated with the concentrations of nutlin-3a indicated in Fig. S3 for 1 h. Infections were carried out normally, without the addition of nutlin-3a. Following removal of the inoculum after absorption, the low-serum medium added back contained the concentration of nutlin-3a used in the pretreatment step. Cells were incubated in low-serum medium supplemented with etoposide (Sigma) at a final concentration of 20 μM for the periods of time indicated in Fig. 2D and Fig. 3D.

### Immunoblots.

Immunoblots were performed as described previously (57) with minor modifications. Briefly, cells were lysed in 1× NuPAGE LDS sample buffer (Invitrogen) containing 5% (vol/vol) 2-mercaptoethanol and a protease/phosphatase inhibitor cocktail (HALT; Thermo Fisher Scientific). Lysates were separated through 4 to 12% gradient NuPAGE bis-Tris gels (Invitrogen) and transferred to nitrocellulose membranes (Bio-Rad). Transfer quality was determined via staining with a solution consisting of 0.5% (wt/vol) Ponceau S (Sigma) and 1% (vol/vol) acetic acid. Stain was removed by washing with phosphate-buffered saline (PBS) containing 0.1% (vol/vol) Tween (PBST). Destained membranes were blocked with a solution of 5% (wt/vol) nonfat milk in PBST for 1 h at room temperature. Primary antibodies were used in 5% milk, and blots were incubated overnight at 4°C. Secondary antibodies were also used in 5% milk for 1 h at room temperature. Proteins were detected by exposure to film (Denville). The reagents used are listed in Table 1.

**TABLE 1:** Reagents used for immunoblotting

Reagent^*a*^	Vendor or reference	Catalog no.	Dilution
Anti-GAPDH	Abcam	ab8245	1:5,000
Anti-gB	Abcam	ab6506	1:5,000
Anti-H2AX	Cell Signaling	7631S	1:1,000
Anti-ATM	Abcam	ab78	1:2,000
Anti-Chk2	Cell Signaling	3440S	1:1,000
Anti-p53 (DO-1)	Santa Cruz Biotech	sc-126	1:1,000
Anti-γH2AX (S139)	Abcam	ab2893	1:2,000
Anti-phospho-ATM (S1981)	Cell Signaling	4526S	1:1,000
Anti-phospho-Chk2 (T68)	Cell Signaling	2197S	1:1,000
Anti-phospho-p53 (S15)	Cell Signaling	9284S	1:1,000
Anti-phospho-p53 (S20)	Invitrogen	PA5-17894	1:1,000
Anti-ICP0	East Coast Bio	H1A027	1:2,000
Anti-Mdc1	Abcam	ab11171	1:2,000
Anti-ICP8	93		1:5,000
Anti-FLAG	Sigma	F1804	1:2,000
Anti-Mre11	Novus Biologicals	NB100-142	1:2,000
Anti-ICP4	94		1:2,000
Anti-53BP1	Abcam	ab21083	1:1,000
Anti-DNA-PKcs	Abcam	ab70250	1:2,000
Anti-IFI16	Abcam	ab55328	1:1,000
Anti-rabbit HRP conjugated	Cell Signaling	7074S	1:5,000
Anti-mouse HRP conjugated	Cell Signaling	7076S	1:5,000

a HRP, horseradish peroxidase.

### siRNA knockdowns.

siRNA transfections were carried out essentially as described previously (90) with minor modifications. Briefly, 1 × 10^5^ HFFs per well in 12-well plates were transfected with 10 pmol of siRNA per well using Lipofectamine RNAiMAX (Invitrogen) according to the manufacturer’s protocol. Two days later, cells were trypsinized, seeded to a lower density, and used the following day. The siRNAs used are listed in Table 2.

**TABLE 2:** siRNAs used

siRNA	Dharmacon catalog no.
ON-TARGETplus nontargeting pool	D-001810-10-50
ON-TARGETplus TP53 SMARTpool	L-003329-00-0005
ON-TARGETplus TP53BP1 SMARTpool	L-003548-00-0005
ON-TARGETplus PRKDC SMARTpool	L-005030-00-0005

### CRISPR-Cas9 gene knockouts.

Knockout cells were generated as described previously (90) with minor modifications. Briefly, oligonucleotides were purchased from IDT and were cloned into the lentiCRISPR v2 backbone plasmid (91). HEK293T cells were transfected with the lentiCRISPR plasmid along with psPAX2 and pVSVG packaging plasmids. Cell supernatants were collected 2 days later and filtered through a 0.45-μm filter. Low-passage HFFs were overlaid with the filtered supernatant. Polybrene (Santa Cruz Biotechnology) was added to a final concentration of 5 μg/ml. After 24 h, virus was removed and replaced with fresh medium containing 1 μg/ml puromycin (Santa Cruz Biotechnology). Knockout efficiency was validated via immunoblotting roughly 2 weeks later, when the cells had grown to confluence in T150 flasks. The targets and their sequences are listed in Table 3.

**TABLE 3:** CRISPR-Cas9 targets

Target and gRNA no.	Sequence (DNA form in oligonucleotide) (5′-3′)	Reference
ATM, gRNA 2	TGATAGAGCTACAGAACGAA	91
CHEK2, gRNA 1	AAGAAGCCTTAAGACACCCG	91
MDC1, gRNA 1	CCGAATGCCTGACTGCTCTG	91
MRE11, gRNA 3	GTTTGCTGCGTATTAAAGGG	95
MRE11, gRNA 4	GCAATCATGACGATCCCACA	95
H2AFX, gRNA 3	CCGCGGCAAGACTGGCGGCA	95
TP53, gRNA 5	CCATTGTTCAATATCGTCCG	95
IFI16, gRNA 1	GTTCCGAGGTGATGCTGGTT	87

### Nucleic acid extraction, reverse transcription, and quantitative PCR.

Both RNA and DNA were extracted and processed for quantitative PCR (qPCR) as described previously (92), with modifications. Briefly, DNA was used directly for qPCR and RNA was DNase treated (Invitrogen DNA-free kit) and subsequently reverse transcribed (Agilent Technologies high-capacity reverse transcription kit), both according to the manufacturer’s suggested protocol, and then used for qPCR. Transcripts were quantified using a standard curve generated by using a 10-fold dilution series of DNA/cDNA prepared from HFFs infected with WT virus. All qPCRs were performed using an Applied Biosystems 7500 Fast real-time PCR system. Fast SYBR green master mix (Applied Biosystems) was used for all qPCRs. The oligonucleotides used are listed in Table 4.

**TABLE 4:** Oligonucleotides used for qPCR

Oligonucleotide	Sequence (5′-3′)	Reference
UL29 forward (DNA)	GAGACCGGGGTTGGGGAATGAATC	64
UL29 reverse (DNA)	CCCGGGGGTTGTCTGTGAAGG	64
GAPDH forward (DNA)	CAGGCGCCCAATACGACCAAATC	87
GAPDH reverse (DNA)	TTCGACAGTCAGCCGCATCTTCTT	87
18S forward (cDNA)	GCATTCGTATTGCGCCGCTA	64
18S reverse (cDNA)	AGCTGCCCGGCGGGTC	64
ICP4 forward (cDNA)	CGGTGATGAAGGAGCTGCTGTTGC	96
ICP4 reverse (cDNA)	CTGATCACGCGGCTGCTGTACA	96
ICP27 forward (cDNA)	AGACGCCTCGTCCGACGGA	96
ICP27 reverse (cDNA)	GAGGCGCGACCACACACTGT	96
ICP8 forward (cDNA)	CATCAGCTGCTCCACCTCGCG	96
ICP8 reverse (cDNA)	GCAGTACGTGGACCAGGCGGT	96
gC forward (cDNA)	GAGGAGGTCCTGACGAACATCACC	96
gC reverse (cDNA)	CCGGTGACAGAATACAACGGAGG	96

### Software for graphs and diagrams.

Graphs and statistical analyses were generated using GraphPad Prism. Figures 6B and 7 were created with Biorender.com.

## References

[1] Lamarche BJ, Orazio NI, Weitzman MD 2010 The MRN complex in double-strand break repair and telomere maintenance. FEBS Lett 584:3682–3695. doi:10.1016/j.febslet.2010.07.029. 20655309 PMC2946096

[2] You Z, Chahwan C, Bailis J, Hunter T, Russell P 2005 ATM activation and its recruitment to damaged DNA require binding to the C terminus of Nbs1. Mol Cell Biol 25:5363–5379. doi:10.1128/MCB.25.13.5363-5379.2005. 15964794 PMC1156989

[3] Burma S, Chen BP, Murphy M, Kurimasa A, Chen DJ 2001 ATM phosphorylates histone H2AX in response to DNA double-strand breaks. J Biol Chem 276:42462–42467. doi:10.1074/jbc.C100466200. 11571274

[4] Stucki M, Clapperton JA, Mohammad D, Yaffe MB, Smerdon SJ, Jackson SP 2005 MDC1 directly binds phosphorylated histone H2AX to regulate cellular responses to DNA double-strand breaks. Cell 123:1213–1226. doi:10.1016/j.cell.2005.09.038. 16377563

[5] Stewart GS, Wang B, Bignell CR, Taylor AM, Elledge SJ 2003 MDC1 is a mediator of the mammalian DNA damage checkpoint. Nature 421:961–966. doi:10.1038/nature01446. 12607005

[6] Kolas NK, Chapman JR, Nakada S, Ylanko J, Chahwan R, Sweeney FD, Panier S, Mendez M, Wildenhain J, Thomson TM, Pelletier L, Jackson SP, Durocher D 2007 Orchestration of the DNA-damage response by the RNF8 ubiquitin ligase. Science 318:1637–1640. doi:10.1126/science.1150034. 18006705 PMC2430610

[7] Doil C, Mailand N, Bekker-Jensen S, Menard P, Larsen DH, Pepperkok R, Ellenberg J, Panier S, Durocher D, Bartek J, Lukas J, Lukas C 2009 RNF168 binds and amplifies ubiquitin conjugates on damaged chromosomes to allow accumulation of repair proteins. Cell 136:435–446. doi:10.1016/j.cell.2008.12.041. 19203579

[8] Mimori T, Hardin JA 1986 Mechanism of interaction between Ku protein and DNA. J Biol Chem 261:10375–10379. doi:10.1016/S0021-9258(18)67534-9. 3015926

[9] Gottlieb TM, Jackson SP 1993 The DNA-dependent protein kinase: requirement for DNA ends and association with Ku antigen. Cell 72:131–142. doi:10.1016/0092-8674(93)90057-w. 8422676

[10] Graham TG, Walter JC, Loparo JJ 2016 Two-stage synapsis of DNA ends during non-homologous end joining. Mol Cell 61:850–858. doi:10.1016/j.molcel.2016.02.010. 26990988 PMC4799494

[11] Critchlow SE, Bowater RP, Jackson SP 1997 Mammalian DNA double-strand break repair protein XRCC4 interacts with DNA ligase IV. Curr Biol 7:588–598. doi:10.1016/s0960-9822(06)00258-2. 9259561

[12] Grawunder U, Wilm M, Wu X, Kulesza P, Wilson TE, Mann M, Lieber MR 1997 Activity of DNA ligase IV stimulated by complex formation with XRCC4 protein in mammalian cells. Nature 388:492–495. doi:10.1038/41358. 9242410

[13] Hafner A, Bulyk ML, Jambhekar A, Lahav G 2019 The multiple mechanisms that regulate p53 activity and cell fate. Nat Rev Mol Cell Biol 20:199–210. doi:10.1038/s41580-019-0110-x. 30824861

[14] Canman CE, Lim DS, Cimprich KA, Taya Y, Tamai K, Sakaguchi K, Appella E, Kastan MB, Siliciano JD 1998 Activation of the ATM kinase by ionizing radiation and phosphorylation of p53. Science 281:1677–1679. doi:10.1126/science.281.5383.1677. 9733515

[15] Tibbetts RS, Brumbaugh KM, Williams JM, Sarkaria JN, Cliby WA, Shieh SY, Taya Y, Prives C, Abraham RT 1999 A role for ATR in the DNA damage-induced phosphorylation of p53. Genes Dev 13:152–157. doi:10.1101/gad.13.2.152. 9925639 PMC316393

[16] Hirao A, Kong YY, Matsuoka S, Wakeham A, Ruland J, Yoshida H, Liu D, Elledge SJ, Mak TW 2000 DNA damage-induced activation of p53 by the checkpoint kinase Chk2. Science 287:1824–1827. doi:10.1126/science.287.5459.1824. 10710310

[17] Loughery J, Cox M, Smith LM, Meek DW 2014 Critical role for p53-serine 15 phosphorylation in stimulating transactivation at p53-responsive promoters. Nucleic Acids Res 42:7666–7680. doi:10.1093/nar/gku501. 24928858 PMC4081099

[18] Dumaz N, Milne DM, Jardine LJ, Meek DW 2001 Critical roles for the serine 20, but not the serine 15, phosphorylation site and for the polyproline domain in regulating p53 turnover. Biochem J 359:459–464. doi:10.1042/bj3590459. 11583595 PMC1222167

[19] Anacker DC, Gautam D, Gillespie KA, Chappell WH, Moody CA 2014 Productive replication of human papillomavirus 31 requires DNA repair factor Nbs1. J Virol 88:8528–8544. doi:10.1128/JVI.00517-14. 24850735 PMC4135936

[20] Moody CA, Laimins LA 2009 Human papillomaviruses activate the ATM DNA damage pathway for viral genome amplification upon differentiation. PLoS Pathog 5:e1000605. doi:10.1371/journal.ppat.1000605. 19798429 PMC2745661

[21] Stracker TH, Carson CT, Weitzman MD 2002 Adenovirus oncoproteins inactivate the Mre11-Rad50-NBS1 DNA repair complex. Nature 418:348–352. doi:10.1038/nature00863. 12124628

[22] Shah GA, O'Shea CC 2015 Viral and cellular genomes activate distinct DNA damage responses. Cell 162:987–1002. doi:10.1016/j.cell.2015.07.058. 26317467 PMC4681434

[23] Hau PM, Tsao SW 2017 Epstein-Barr virus hijacks DNA damage response transducers to orchestrate its life cycle. Viruses 9:341. doi:10.3390/v9110341. PMC5707548 29144413

[24] Hollingworth R, Horniblow RD, Forrest C, Stewart GS, Grand RJ 2017 Localization of double-strand break repair proteins to viral replication compartments following lytic reactivation of Kaposi's sarcoma-associated herpesvirus. J Virol 91:e00930-17. doi:10.1128/JVI.00930-17. 28855246 PMC5660498

[25] E X, Pickering MT, Debatis M, Castillo J, Lagadinos A, Wang S, Lu S, Kowalik TF 2011 An E2F1-mediated DNA damage response contributes to the replication of human cytomegalovirus. PLoS Pathog 7:e1001342. doi:10.1371/journal.ppat.1001342. 21589897 PMC3093362

[26] Roizman B, Knipe DM, Whitley RJ 2013 Herpes simplex viruses, p 1823–1897. *In* Knipe DM, Howley PM, Cohen JI, Griffin DE, Lamb RA, Martin MA, Racaniello VR, Roizman B (ed), Fields virology, 6th ed Lippincott Williams & Wilkins, Philadelphia, PA.

[27] Orzalli MH, DeLuca NA, Knipe DM 2012 Nuclear IFI16 induction of IRF-3 signaling during herpesviral infection and degradation of IFI16 by the viral ICP0 protein. Proc Natl Acad Sci U S A 109:E3008–E3017. doi:10.1073/pnas.1211302109. 23027953 PMC3497734

[28] Chelbi-Alix MK, de Thé H 1999 Herpes virus induced proteasome-dependent degradation of the nuclear bodies-associated PML and Sp100 proteins. Oncogene 18:935–941. doi:10.1038/sj.onc.1202366. 10023669

[29] Jurak I, Silverstein LB, Sharma M, Coen DM 2012 Herpes simplex virus is equipped with RNA- and protein-based mechanisms to repress expression of ATRX, an effector of intrinsic immunity. J Virol 86:10093–10102. doi:10.1128/JVI.00930-12. 22787211 PMC3446562

[30] Lukashchuk V, Everett RD 2010 Regulation of ICP0-null mutant herpes simplex virus type 1 infection by ND10 components ATRX and hDaxx. J Virol 84:4026–4040. doi:10.1128/JVI.02597-09. 20147399 PMC2849514

[31] Gu H, Roizman B 2003 The degradation of promyelocytic leukemia and Sp100 proteins by herpes simplex virus 1 is mediated by the ubiquitin-conjugating enzyme UbcH5a. Proc Natl Acad Sci U S A 100:8963–8968. doi:10.1073/pnas.1533420100. 12855769 PMC166421

[32] Lilley CE, Carson CT, Muotri AR, Gage FH, Weitzman MD 2005 DNA repair proteins affect the lifecycle of herpes simplex virus 1. Proc Natl Acad Sci U S A 102:5844–5849. doi:10.1073/pnas.0501916102. 15824307 PMC556126

[33] Lilley CE, Chaurushiya MS, Boutell C, Everett RD, Weitzman MD 2011 The intrinsic antiviral defense to incoming HSV-1 genomes includes specific DNA repair proteins and is counteracted by the viral protein ICP0. PLoS Pathog 7:e1002084. doi:10.1371/journal.ppat.1002084. 21698222 PMC3116817

[34] Taylor TJ, Knipe DM 2004 Proteomics of herpes simplex virus replication compartments: association of cellular DNA replication, repair, recombination, and chromatin remodeling proteins with ICP8. J Virol 78:5856–5866. doi:10.1128/JVI.78.11.5856-5866.2004. 15140983 PMC415816

[35] Alekseev O, Donovan K, Azizkhan-Clifford J 2014 Inhibition of ataxia telangiectasia mutated (ATM) kinase suppresses herpes simplex virus type 1 (HSV-1) keratitis. Invest Ophthalmol Vis Sci 55:706–715. doi:10.1167/iovs.13-13461. 24370835 PMC3912898

[36] Botting C, Lu X, Triezenberg SJ 2016 H2AX phosphorylation and DNA damage kinase activity are dispensable for herpes simplex virus replication. Virol J 13:15. doi:10.1186/s12985-016-0470-1. 26817608 PMC4728825

[37] Gregory DA, Bachenheimer SL 2008 Characterization of mre11 loss following HSV-1 infection. Virology 373:124–136. doi:10.1016/j.virol.2007.12.005. 18177684 PMC2295170

[38] Edwards TG, Bloom DC, Fisher C 2017 The ATM and Rad3-related (ATR) protein kinase pathway is activated by herpes simplex virus 1 and required for efficient viral replication. J Virol 92:e01884-17. doi:10.1128/JVI.01884-17. PMC5827400 29263259

[39] Wilkinson DE, Weller SK 2006 Herpes simplex virus type I disrupts the ATR-dependent DNA-damage response during lytic infection. J Cell Sci 119:2695–2703. doi:10.1242/jcs.02981. 16757521 PMC4427570

[40] Dembowski JA, DeLuca NA 2015 Selective recruitment of nuclear factors to productively replicating herpes simplex virus genomes. PLoS Pathog 11:e1004939. doi:10.1371/journal.ppat.1004939. 26018390 PMC4446364

[41] Dembowski JA, DeLuca NA 2018 Temporal viral genome-protein interactions define distinct stages of productive herpesviral infection. mBio 9:e01182-18. doi:10.1128/mBio.01182-18. 30018111 PMC6050965

[42] Wilcock D, Lane DP 1991 Localization of p53, retinoblastoma and host replication proteins at sites of viral replication in herpes-infected cells. Nature 349:429–431. doi:10.1038/349429a0. 1671528

[43] Maruzuru Y, Fujii H, Oyama M, Kozuka-Hata H, Kato A, Kawaguchi Y 2013 Roles of p53 in herpes simplex virus 1 replication. J Virol 87:9323–9332. doi:10.1128/JVI.01581-13. 23785201 PMC3754062

[44] Maruzuru Y, Koyanagi N, Takemura N, Uematsu S, Matsubara D, Suzuki Y, Arii J, Kato A, Kawaguchi Y 2016 p53 is a host cell regulator during herpes simplex encephalitis. J Virol 90:6738–6745. doi:10.1128/JVI.00846-16. 27170756 PMC4944289

[45] Samaniego LA, Neiderhiser L, DeLuca NA 1998 Persistence and expression of the herpes simplex virus genome in the absence of immediate-early proteins. J Virol 72:3307–3320. doi:10.1128/JVI.72.4.3307-3320.1998. 9525658 PMC109808

[46] Honess RW, Roizman B 1974 Regulation of herpesvirus macromolecular synthesis. I. Cascade regulation of the synthesis of three groups of viral proteins. J Virol 14:8–19. doi:10.1128/JVI.14.1.8-19.1974. 4365321 PMC355471

[47] Kang MA, So EY, Simons AL, Spitz DR, Ouchi T 2012 DNA damage induces reactive oxygen species generation through the H2AX-Nox1/Rac1 pathway. Cell Death Dis 3:e249. doi:10.1038/cddis.2011.134. 22237206 PMC3270268

[48] Li H, Baskaran R, Krisky DM, Bein K, Grandi P, Cohen JB, Glorioso JC 2008 Chk2 is required for HSV-1 ICP0-mediated G2/M arrest and enhancement of virus growth. Virology 375:13–23. doi:10.1016/j.virol.2008.01.038. 18321553 PMC2706573

[49] Cai WZ, Schaffer PA 1989 Herpes simplex virus type 1 ICP0 plays a critical role in the de novo synthesis of infectious virus following transfection of viral DNA. J Virol 63:4579–4589. doi:10.1128/JVI.63.11.4579-4589.1989. 2552142 PMC251091

[50] An J, Huang YC, Xu QZ, Zhou LJ, Shang ZF, Huang B, Wang Y, Liu XD, Wu DC, Zhou PK 2010 DNA-PKcs plays a dominant role in the regulation of H2AX phosphorylation in response to DNA damage and cell cycle progression. BMC Mol Biol 11:18. doi:10.1186/1471-2199-11-18. 20205745 PMC2844398

[51] Mukherjee B, Kessinger C, Kobayashi J, Chen BP, Chen DJ, Chatterjee A, Burma S 2006 DNA-PK phosphorylates histone H2AX during apoptotic DNA fragmentation in mammalian cells. DNA Repair (Amst) 5:575–590. doi:10.1016/j.dnarep.2006.01.011. 16567133

[52] Ward IM, Chen J 2001 Histone H2AX is phosphorylated in an ATR-dependent manner in response to replicational stress. J Biol Chem 276:47759–47762. doi:10.1074/jbc.C100569200. 11673449

[53] Lees-Miller SP, Long MC, Kilvert MA, Lam V, Rice SA, Spencer CA 1996 Attenuation of DNA-dependent protein kinase activity and its catalytic subunit by the herpes simplex virus type 1 transactivator ICP0. J Virol 70:7471–7477. doi:10.1128/JVI.70.11.7471-7477.1996. 8892865 PMC190814

[54] Parkinson J, Lees-Miller SP, Everett RD 1999 Herpes simplex virus type 1 immediate-early protein vmw110 induces the proteasome-dependent degradation of the catalytic subunit of DNA-dependent protein kinase. J Virol 73:650–657. doi:10.1128/JVI.73.1.650-657.1999. 9847370 PMC103871

[55] Boutell C, Everett RD 2004 Herpes simplex virus type 1 infection induces the stabilization of p53 in a USP7- and ATM-independent manner. J Virol 78:8068–8077. doi:10.1128/JVI.78.15.8068-8077.2004. 15254178 PMC446092

[56] Vassilev LT, Vu BT, Graves B, Carvajal D, Podlaski F, Filipovic Z, Kong N, Kammlott U, Lukacs C, Klein C, Fotouhi N, Liu EA 2004 In vivo activation of the p53 pathway by small-molecule antagonists of MDM2. Science 303:844–848. doi:10.1126/science.1092472. 14704432

[57] Orzalli MH, Conwell SE, Berrios C, DeCaprio JA, Knipe DM 2013 Nuclear interferon-inducible protein 16 promotes silencing of herpesviral and transfected DNA. Proc Natl Acad Sci U S A 110:E4492–E4501. doi:10.1073/pnas.1316194110. 24198334 PMC3839728

[58] Johnstone RW, Wei W, Greenway A, Trapani JA 2000 Functional interaction between p53 and the interferon-inducible nucleoprotein IFI 16. Oncogene 19:6033–6042. doi:10.1038/sj.onc.1204005. 11146555

[59] Liao JC, Lam R, Brazda V, Duan S, Ravichandran M, Ma J, Xiao T, Tempel W, Zuo X, Wang YX, Chirgadze NY, Arrowsmith CH 2011 Interferon-inducible protein 16: insight into the interaction with tumor suppressor p53. Structure 19:418–429. doi:10.1016/j.str.2010.12.015. 21397192 PMC3760383

[60] Kwak JC, Ongusaha PP, Ouchi T, Lee SW 2003 IFI16 as a negative regulator in the regulation of p53 and p21(Waf1). J Biol Chem 278:40899–40904. doi:10.1074/jbc.M308012200. 12925527

[61] Fujiuchi N, Aglipay JA, Ohtsuka T, Maehara N, Sahin F, Su GH, Lee SW, Ouchi T 2004 Requirement of IFI16 for the maximal activation of p53 induced by ionizing radiation. J Biol Chem 279:20339–20344. doi:10.1074/jbc.M400344200. 14990579

[62] Smith S, Reuven N, Mohni KN, Schumacher AJ, Weller SK 2014 Structure of the herpes simplex virus 1 genome: manipulation of nicks and gaps can abrogate infectivity and alter the cellular DNA damage response. J Virol 88:10146–10156. doi:10.1128/JVI.01723-14. 24965466 PMC4136335

[63] Rogakou EP, Boon C, Redon C, Bonner WM 1999 Megabase chromatin domains involved in DNA double-strand breaks in vivo. J Cell Biol 146:905–916. doi:10.1083/jcb.146.5.905. 10477747 PMC2169482

[64] Lee JS, Raja P, Knipe DM 2016 Herpesviral ICP0 protein promotes two waves of heterochromatin removal on an early viral promoter during lytic infection. mBio 7:e02007-15. doi:10.1128/mBio.02007-15. PMC4725016 26758183

[65] Dembowski JA, Dremel SE, DeLuca NA 2017 Replication-coupled recruitment of viral and cellular factors to herpes simplex virus type 1 replication forks for the maintenance and expression of viral genomes. PLoS Pathog 13:e1006166. doi:10.1371/journal.ppat.1006166. 28095497 PMC5271410

[66] Balasubramanian N, Bai P, Buchek G, Korza G, Weller SK 2010 Physical interaction between the herpes simplex virus type 1 exonuclease, UL12, and the DNA double-strand break-sensing MRN complex. J Virol 84:12504–12514. doi:10.1128/JVI.01506-10. 20943970 PMC3004347

[67] Conley AJ, Knipe DM, Jones PC, Roizman B 1981 Molecular genetics of herpes simplex virus. VII. Characterization of a temperature-sensitive mutant produced by in vitro mutagenesis and defective in DNA synthesis and accumulation of gamma polypeptides. J Virol 37:191–206. doi:10.1128/JVI.37.1.191-206.1981. 6260973 PMC170996

[68] Theard D, Coisy M, Ducommun B, Concannon P, Darbon JM 2001 Etoposide and adriamycin but not genistein can activate the checkpoint kinase Chk2 independently of ATM/ATR. Biochem Biophys Res Commun 289:1199–1204. doi:10.1006/bbrc.2001.6095. 11741320

[69] Pabla N, Huang S, Mi QS, Daniel R, Dong Z 2008 ATR-Chk2 signaling in p53 activation and DNA damage response during cisplatin-induced apoptosis. J Biol Chem 283:6572–6583. doi:10.1074/jbc.M707568200. 18162465

[70] Li J, Stern DF 2005 Regulation of CHK2 by DNA-dependent protein kinase. J Biol Chem 280:12041–12050. doi:10.1074/jbc.M412445200. 15668230

[71] Kelly BJ, Fraefel C, Cunningham AL, Diefenbach RJ 2009 Functional roles of the tegument proteins of herpes simplex virus type 1. Virus Res 145:173–186. doi:10.1016/j.virusres.2009.07.007. 19615419

[72] Gibson W, Roizman B 1971 Compartmentalization of spermine and spermidine in the herpes simplex virion. Proc Natl Acad Sci U S A 68:2818–2821. doi:10.1073/pnas.68.11.2818. 5288261 PMC389533

[73] Kent JR, Zeng PY, Atanasiu D, Gardner J, Fraser NW, Berger SL 2004 During lytic infection herpes simplex virus type 1 is associated with histones bearing modifications that correlate with active transcription. J Virol 78:10178–10186. doi:10.1128/JVI.78.18.10178-10186.2004. 15331750 PMC514973

[74] Oh J, Fraser NW 2008 Temporal association of the herpes simplex virus genome with histone proteins during a lytic infection. J Virol 82:3530–3537. doi:10.1128/JVI.00586-07. 18160436 PMC2268451

[75] Sun Y, Jiang X, Chen S, Fernandes N, Price BD 2005 A role for the Tip60 histone acetyltransferase in the acetylation and activation of ATM. Proc Natl Acad Sci U S A 102:13182–13187. doi:10.1073/pnas.0504211102. 16141325 PMC1197271

[76] Chailleux C, Tyteca S, Papin C, Boudsocq F, Puget N, Courilleau C, Grigoriev M, Canitrot Y, Trouche D 2010 Physical interaction between the histone acetyl transferase Tip60 and the DNA double-strand breaks sensor MRN complex. Biochem J 426:365–371. doi:10.1042/BJ20091329. 20070254

[77] Li R, Zhu J, Xie Z, Liao G, Liu J, Chen MR, Hu S, Woodard C, Lin J, Taverna SD, Desai P, Ambinder RF, Hayward GS, Qian J, Zhu H, Hayward SD 2011 Conserved herpesvirus kinases target the DNA damage response pathway and TIP60 histone acetyltransferase to promote virus replication. Cell Host Microbe 10:390–400. doi:10.1016/j.chom.2011.08.013. 22018239 PMC3253558

[78] Saffert RT, Kalejta RF 2008 Promyelocytic leukemia-nuclear body proteins: herpesvirus enemies, accomplices, or both? Future Virol 3:265–277. doi:10.2217/17460794.3.3.265. 19763230 PMC2744987

[79] Dellaire G, Ching RW, Ahmed K, Jalali F, Tse KC, Bristow RG, Bazett-Jones DP 2006 Promyelocytic leukemia nuclear bodies behave as DNA damage sensors whose response to DNA double-strand breaks is regulated by NBS1 and the kinases ATM, Chk2, and ATR. J Cell Biol 175:55–66. doi:10.1083/jcb.200604009. 17030982 PMC2064496

[80] Orzalli MH, Broekema NM, Knipe DM 2016 Relative contributions of herpes simplex virus 1 ICP0 and vhs to loss of cellular IFI16 vary in different human cell types. J Virol 90:8351–8359. doi:10.1128/JVI.00939-16. 27412599 PMC5008076

[81] Tang KW, Norberg P, Holmudden M, Elias P, Liljeqvist J 2014 Rad51 and Rad52 are involved in homologous recombination of replicating herpes simplex virus DNA. PLoS One 9:e111584. doi:10.1371/journal.pone.0111584. 25365323 PMC4218770

[82] Muylaert I, Elias P 2010 Contributions of nucleotide excision repair, DNA polymerase eta, and homologous recombination to replication of UV-irradiated herpes simplex virus type 1. J Biol Chem 285:13761–13768. doi:10.1074/jbc.M110.107920. 20215648 PMC2859539

[83] Boutell C, Everett RD 2003 The herpes simplex virus type 1 (HSV-1) regulatory protein ICP0 interacts with and ubiquitinates p53. J Biol Chem 278:36596–36602. doi:10.1074/jbc.M300776200. 12855695

[84] Hsieh JC, Kuta R, Armour CR, Boehmer PE 2014 Identification of two novel functional p53 responsive elements in the herpes simplex virus-1 genome. Virology 460-461:45–54. doi:10.1016/j.virol.2014.04.019. 25010269 PMC4090806

[85] Brown JC 2017 Herpes simplex virus latency: the DNA repair-centered pathway. Adv Virol 2017:7028194. doi:10.1155/2017/7028194. 28255301 PMC5309397

[86] Hu HL, Shiflett LA, Kobayashi M, Chao MV, Wilson AC, Mohr I, Huang TT 2019 TOP2beta-dependent nuclear DNA damage shapes extracellular growth factor responses via dynamic AKT phosphorylation to control virus latency. Mol Cell 74:466–480.E4. doi:10.1016/j.molcel.2019.02.032. 30930055 PMC6499694

[87] Merkl PE, Orzalli MH, Knipe DM 2018 Mechanisms of host IFI16, PML, and Daxx protein restriction of herpes simplex virus 1 replication. J Virol 92:e00057-18. doi:10.1128/JVI.00057-18. 29491153 PMC5923075

[88] Schaffer P, Vonka V, Lewis R, Benyesh-Melnick M 1970 Temperature-sensitive mutants of herpes simplex virus. Virology 42:1144–1146. doi:10.1016/0042-6822(70)90364-8. 4321307

[89] Oh HS, Neuhausser WM, Eggan P, Angelova M, Kirchner R, Eggan KC, Knipe DM 2019 Herpesviral lytic gene functions render the viral genome susceptible to novel editing by CRISPR/Cas9. Elife 8:e51662. doi:10.7554/eLife.51662. 31789594 PMC6917492

[90] Cabral JM, Oh HS, Knipe DM 2018 ATRX promotes maintenance of herpes simplex virus heterochromatin during chromatin stress. Elife 7:e40228. doi:10.7554/eLife.40228. 30465651 PMC6307862

[91] Sanjana NE, Shalem O, Zhang F 2014 Improved vectors and genome-wide libraries for CRISPR screening. Nat Methods 11:783–784. doi:10.1038/nmeth.3047. 25075903 PMC4486245

[92] Raja P, Lee JS, Pan D, Pesola JM, Coen DM, Knipe DM 2016 A herpesviral lytic protein regulates the structure of latent viral chromatin. mBio 7:e00633-16. doi:10.1128/mBio.00633-16. 27190217 PMC4895110

[93] Knipe DM, Senechek D, Rice SA, Smith JL 1987 Stages in the nuclear association of the herpes simplex virus transcriptional activator protein ICP4. J Virol 61:276–284. doi:10.1128/JVI.61.2.276-284.1987. 3027360 PMC253947

[94] Showalter SD, Zweig M, Hampar B 1981 Monoclonal antibodies to herpes simplex virus type 1 proteins, including the immediate-early protein ICP 4. Infect Immun 34:684–692. doi:10.1128/IAI.34.3.684-692.1981. 6277788 PMC350925

[95] Doench JG, Fusi N, Sullender M, Hegde M, Vaimberg EW, Donovan KF, Smith I, Tothova Z, Wilen C, Orchard R, Virgin HW, Listgarten J, Root DE 2016 Optimized sgRNA design to maximize activity and minimize off-target effects of CRISPR-Cas9. Nat Biotechnol 34:184–191. doi:10.1038/nbt.3437. 26780180 PMC4744125

[96] Garvey CE, McGowin CL, Foster TP 2014 Development and evaluation of SYBR green-I based quantitative PCR assays for herpes simplex virus type 1 whole transcriptome analysis. J Virol Methods 201:101–111. doi:10.1016/j.jviromet.2014.02.010. 24607486 PMC4041175

