# Supplementary figures and images for: Herpes Simplex Virus 1 Manipulates Host Cell Antiviral and Proviral DNA Damage Responses
Source: mBio. 2021 Feb 9;12(1):e03552-20. doi: 10.1128/mBio.03552-20 (PMC7885110; doi:10.1128/mBio.03552-20)

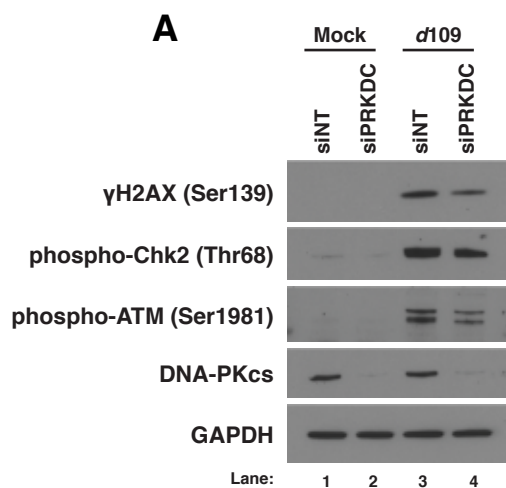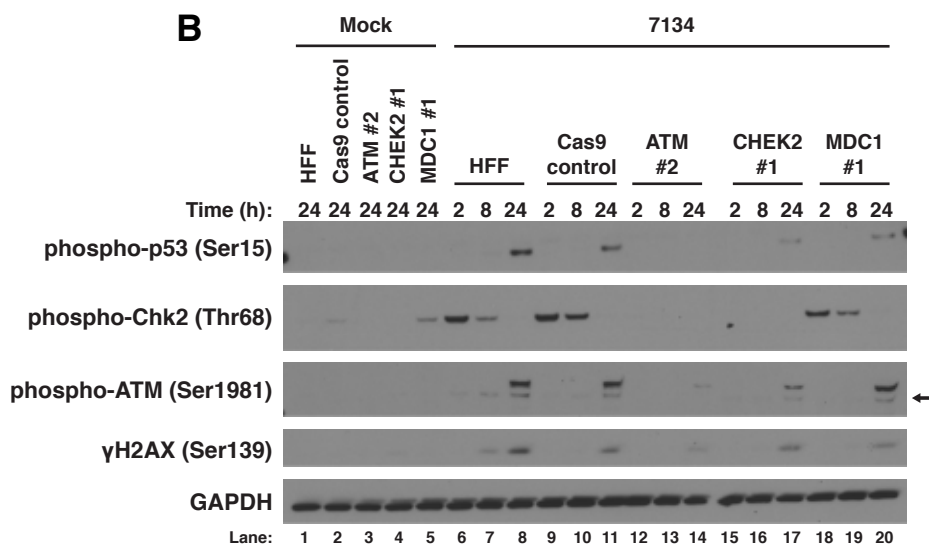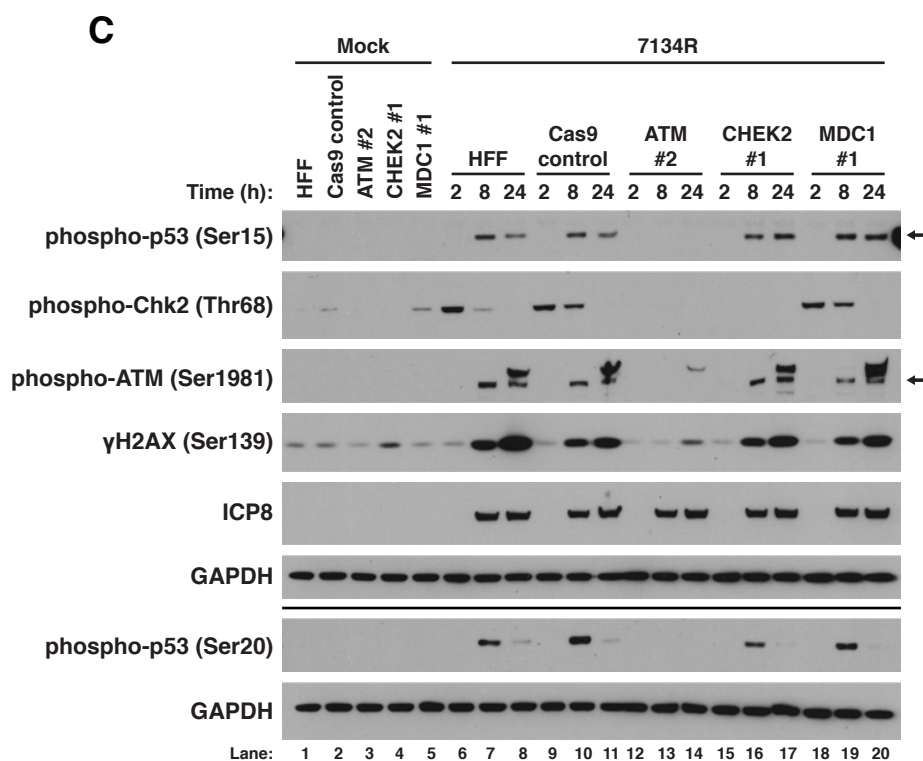

Supplement: FIG S1 [file mBio.03552-20-sf001.pdf]

**A**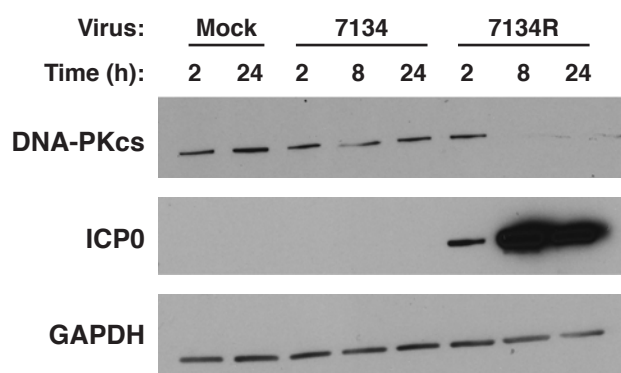**B**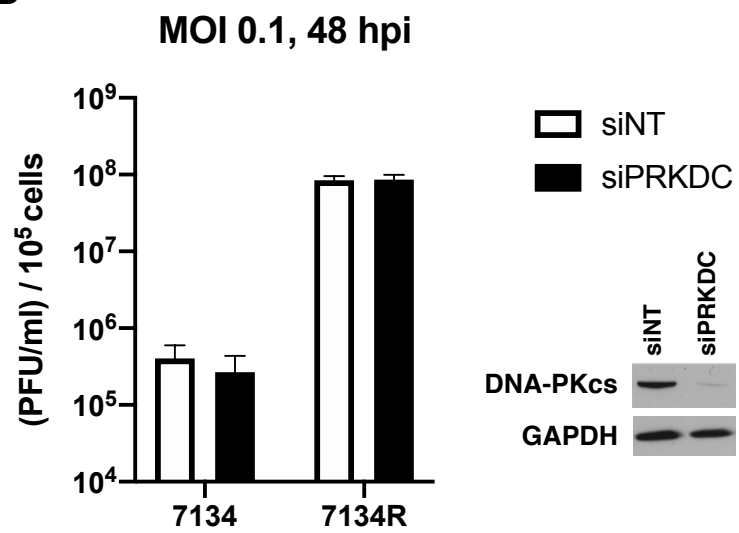

Supplement: FIG S2 [file mBio.03552-20-sf002.pdf]

**A**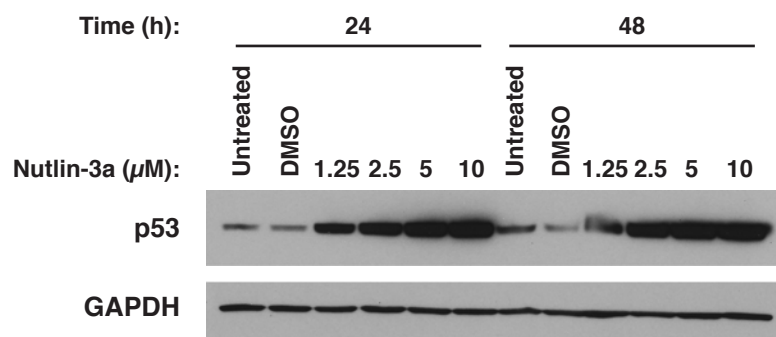**B**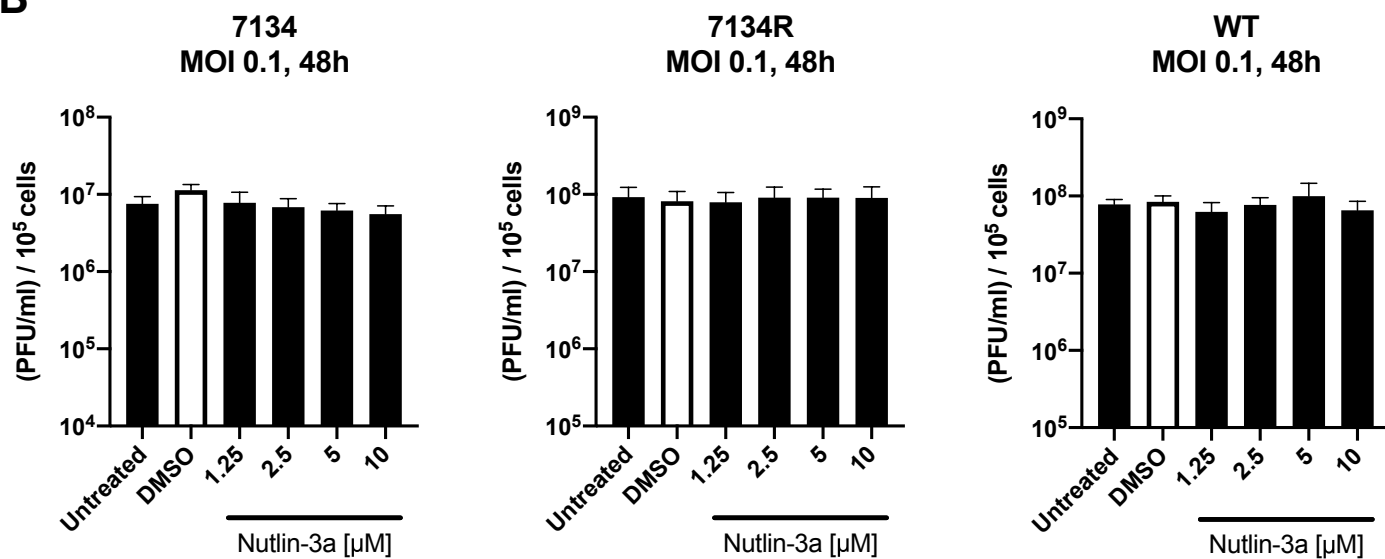

Supplement: FIG S3 [file mBio.03552-20-sf003.pdf]

**A**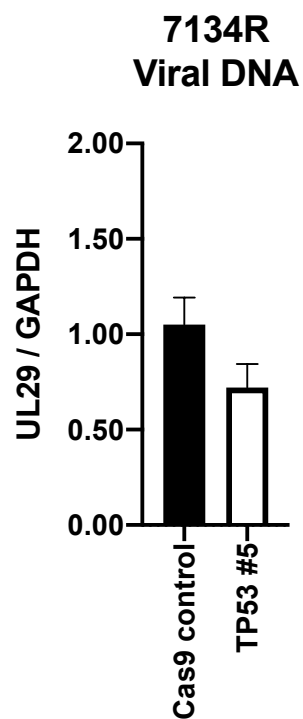**B**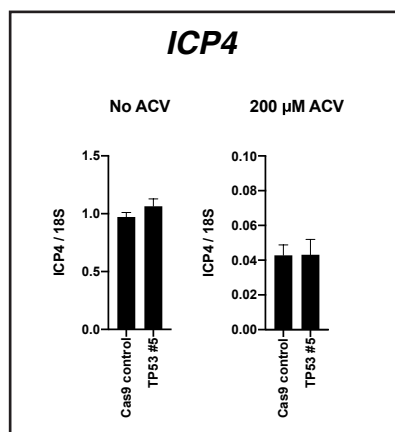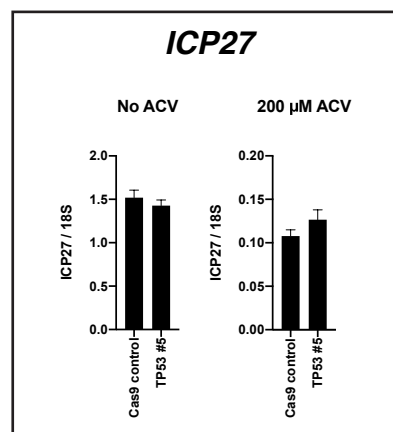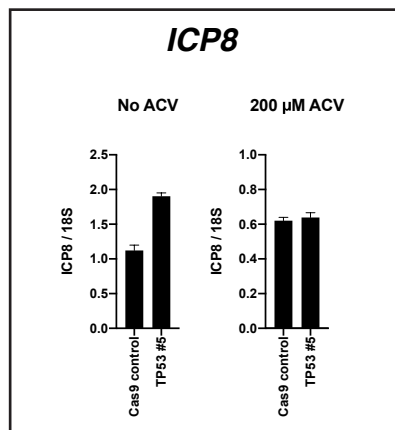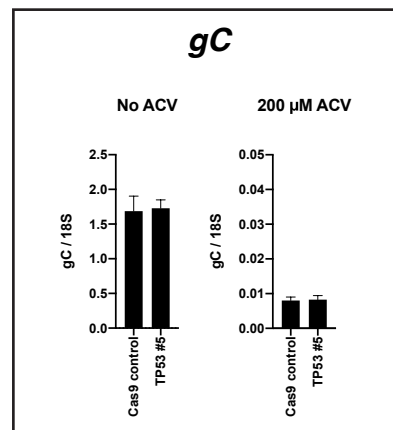**C**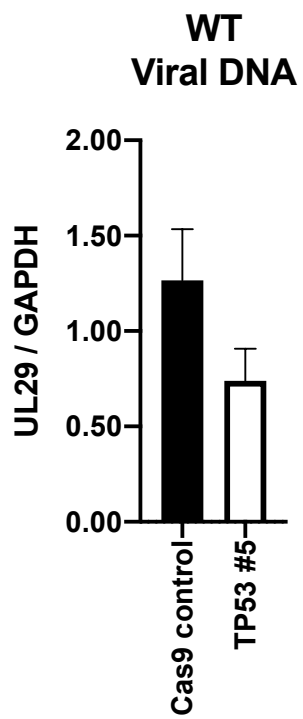**D**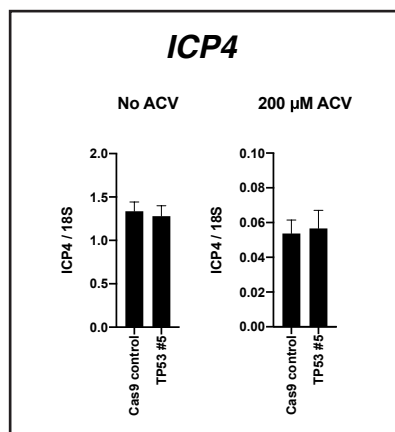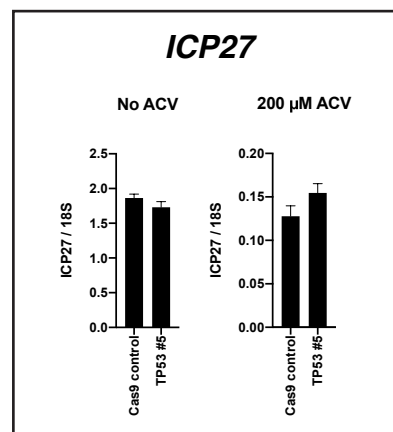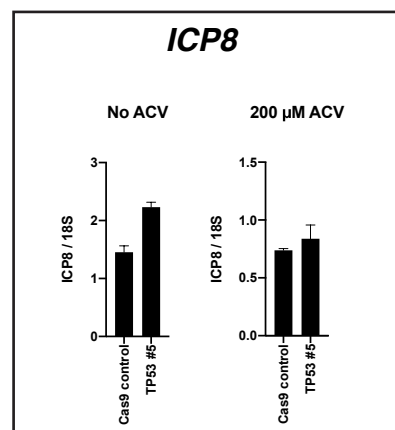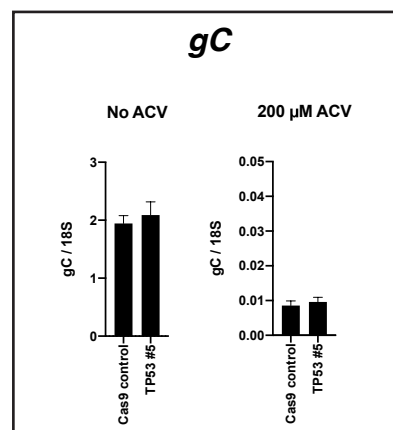

Supplement: FIG S4 [file mBio.03552-20-sf004.pdf]

**A**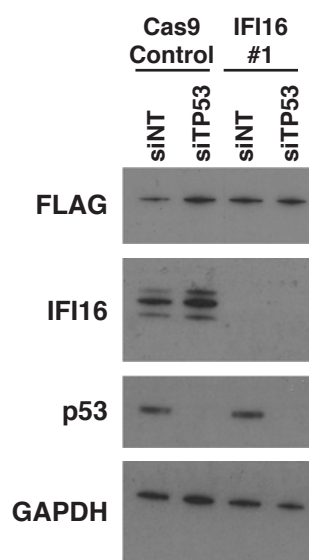**B**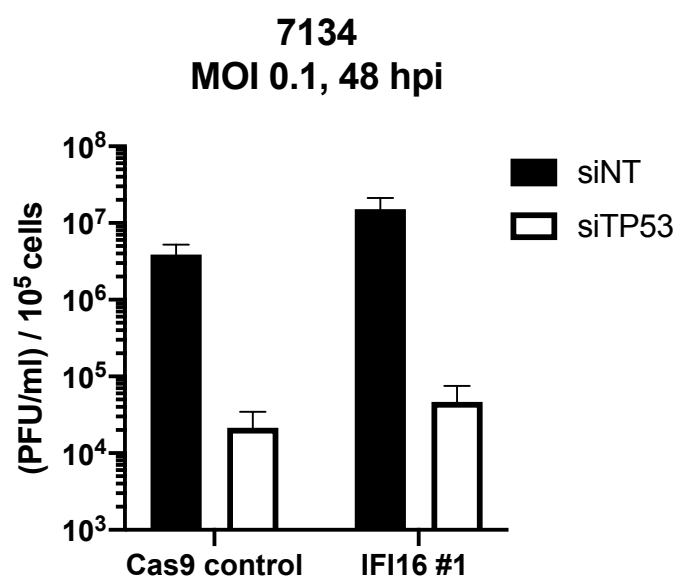

Supplement: FIG S5 [file mBio.03552-20-sf005.pdf]
